# Evaluating the Performance of a Microporous Ti Bisphosphonate MOF for Postcombustion Carbon Capture by Vacuum Pressure Swing Adsorption

**DOI:** 10.1021/acs.iecr.5c00734

**Published:** 2025-07-23

**Authors:** Shreenath Krishnamurthy, Nicolas Heymans, Mohammad Wahidduzzaman, Guillaume Maurin, Shyamapada Nandi, Richard Blom, Debanjan Chakraborty, Farid Nouar, Christian Serre, Giorgia Mondino, Georges Mouchaham, Guy De Weireld

**Affiliations:** † Process Technology, 6298 SINTEF INDUSTRY, 0373 Oslo, Norway; ‡ Thermodynamics and Mathematical Physics Unit, 54521 University of Mons (UMONS), 7000 Mons, Belgium; § 26909 ICGM, University of Montpellier, CNRS, ENSCM, 34293 Montpellier, France; ∥ Institut des Matériaux Poreux de Paris, ENS, ESPCI Paris, CNRS, PSL University, 75005 Paris, France

## Abstract

A multiscale study was carried out to evaluate the microporous -Ti-bisphosphonate MIL-91­(Ti) sorbent for postcombustion CO_2_ capture in industrially relevant conditions. The process performance of the MOF was first assessed by using molecular simulated adsorption isotherms, which predicted an energy consumption of 1.65 MJ/kg and a productivity value of 0.42 mol/m^3^. Subsequently, this Ti-MOF was characterized using several complementary experimental techniques, and the characterization data were supplied to a process simulator to assess energy consumption and productivity values for 95% purity and 90% recovery targets. The experimental adsorption isotherms resulted in a better process performance, with a minimum energy consumption of 1.03 MJ/kg and a maximum productivity of 0.61 mol/m^3^. Such a discrepancy is likely to be due to the use of a generic force field that does not accurately capture host–guest intermolecular interactions in a highly confined environment of ultramicroporous MOFs like MIL-91. However, the lower energy consumption and higher productivity of this MOF, which are both desirable outcomes for CO_2_ capture processes, suggest the viability of MIL-91­(Ti) for implications in real CCS applications.

## Graphical abstract

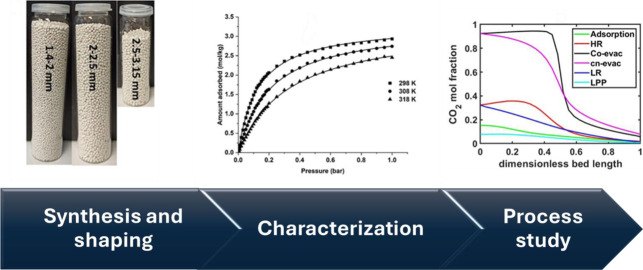

## Introduction

Metal–organic frameworks (MOFs) are a relatively recent group of ordered porous materials comprising a metal cluster (or chains, layers) and organic ligands to form 2D or 3D structures bearing one-dimensional, two-dimensional, and three-dimensional micro- or mesopores. These solids are known to have relatively low densities and high surface areas and are widely studied at low TRL level for several applications, including catalysis,^1–3^ drug delivery,^4–6^ energy storage,^7–9^ gas purification, and gas separation applications, including carbon capture, among others.^10^ Recently, together with their scale-up at the industrial scale, this has led to the first commercializations of MOFs in domains such as CO_2_ capture in flue gases,^11–13^ Direct Air Capture,^14,15^ or the degradation of chemical warfare.^16,17^

One such example of a metal–organic framework of interest is MIL-91­(Ti). It is constructed from piperazine bisphosphonic acid as the organimAc ligand and Titanium as the metal site^18^ (Figure S1). Its inorganic subunit is composed of a corner-sharing chain of TiO_6_ octahedra connected by the bisphosphonate ligand, delimiting narrow elongated channels. Noticeably, one P–OH group from each phosphonate group is pending inside the narrow pores, interacting with the inorganic chain and the N atoms from the piperazine moieties; this makes the micropores highly polar and the MOF rather hydrophilic. Its synthesis can also be achieved under green and ambient pressure conditions, paving the way for an economically viable scale-up. Consequently, this MOF has been reported to exhibit: 1D small micropores (3.5 Å × 3.5 Å), good CO_2_ capacities at low pressure (>1 mol/kg) for postcombustion at 298 K, together with CO_2_/N_2_ selectivity values ranging from 60 to 100 under postcombustion conditions,^19^ as well as an outstanding hydrothermal stability.

Following DOE recommendations, a successful CO_2_ capture process must be able to achieve high purity and recovery (>95% and 90%) with the lowest energy consumption and highest productivity. In the postcombustion carbon capture process, the typical CO_2_ concentration in a powerplant flue gas is between 4 and 15%. Concentrating the flue gas to >95% requires a sorbent that not only has a high CO_2_ capacity and low N_2_ adsorption but also high working capacity. It has been shown earlier that N_2_ affinity plays a key role in determining the purity of the CO_2_ product in a carbon capture process.^20,21^ Additionally, the adsorbent must also have fast kinetics, which would help the process achieve higher productivity. While high CO_2_/N_2_ selectivity and CO_2_ and/or working capacities, low or moderate heat of adsorption are important, they are not indicators of the performance of a given sorbent in a cyclic adsorption process, and the true performance of a sorbent can be determined by rigorous process simulations. The previous studies dealing with MIL-91­(Ti) were mostly restricted to powders,^22^ while in an actual CO_2_ capture process, the sorbent shall be shaped, for instance, in the form of pellets.

The current study is undertaken to address these gaps in the literature. The goal of the present work is as follows:

1. Synthesize, shape, and characterize MIL-91­(Ti) at a few hundred grams scale.
2. Measurement of pure CO_2_ and N_2_ adsorption isotherms on the pellets.
3. Measurement of adsorption kinetics on the MIL-91­(Ti) pellets.
4. Evaluate the performance of MIL-91­(Ti) in a pressure vacuum swing adsorption process

## Materials and Methods

### Synthesis and Shaping of MIL-91

The synthesis of MIL-91­(Ti) was performed, adapted from a previous protocol,^19^ as follows: the reaction was performed using Ti­(O)­(acac)_2_ (1.09 g, 4.17 mmol) and *N*,*N*′-piperazine (bismethylenephosphonic acid) ligand (1.14 g, 4.17 mmol) (provided by SIKEMIA) under reflux in 30 mL of water for 24 h. The large-scale reaction was carried out under similar conditions. At first, the ligand (500 g) was added to a glass reactor, followed by the addition of 13 L of water. The mixture was heated at 353 K under stirring for 30 min, and then the metal precursor (475 g) was added. The mixture was then refluxed for 24 h. After the completion of the reaction, the product was filtered and washed with plenty of water, followed by drying in a vacuum oven for 24 h at 378 K (∼600 g of powder has been obtained). The solid was then characterized by using PXRD, TGA, IR, and porosity analysis.

242.5 g portion of MIL-91­(Ti) was mixed with 7.5 g of PVB binder (3 weight % PVB and isopropanol as solvent). The mixture was then transferred to a granulator and shaped by spraying isopropanol into the mixture. Through this wet granulation process, the MOF was shaped into different size ranges (1.4–2 and 2–2.5 mm).

### Adsorbent Characterization

#### Surface Area and Pore Characterization

Specific surface areas were estimated from N_2_ isotherms recorded at liquid nitrogen (77 K) temperatures by using the BET method. Sample activation was typically carried out overnight at an external pretreatment unit (BELPREP II vac) at 363 K under vacuum before a short (2 h) pretreatment at the BELSORP Max instrument. The micropore volume was estimated using the *t*-plot method based on said N_2_ isotherm measurements at 77 K, while meso- and macropores were analyzed using a Hg porosimeter (Micromeritics AutoPore IV 9520) operating from 0.1 Pa to 414 MPa, covering the pore diameter range from approximately 360 to 3 nm.

#### Crushing Strength Tests

Three to 4 beads having similar shape and size were selected for the compression test in order to minimize the deviation in mechanical stability. The machine used was a Zwick/Roell Z250 universal test machine equipped with a 500 N load cell. One bead at a time was placed between the parallel compression plates. The lower compression plate was raised at a rate of 0.2 mm/min, while the force (in Newtons) was recorded as a function of deformation of the bead in millimeters. The output data was collected using the software TestXpert II. When each particle breaks, there is a sudden decrease in the required force. The force at the breaking point was noted, and results for the 3–4 beads are averaged and reported as the average crushing strength.

### Adsorption Measurements

CO_2_ and N_2_ excess adsorption isotherms (293, 303, and 313 K) were measured in a built-in-house device using a high-pressure magnetic suspension balance (gravimetry) from Rubotherm at UMONS. Before each measurement, the adsorbent (around 2 g) was outgassed at 393 K under a secondary vacuum for 12 h. The adsorbent was then exposed to gases, and the mass variation, the pressure, and the temperature were monitored until equilibrium was reached (criterion: when four of the last five mass measurements (noticed every 5 min) are included in an interval of 50 μg). The buoyancy effect of the gas phase on the adsorbent volume (evaluated by direct helium buoyancy effect measurement) was corrected to determine the excess adsorbed mass. Some isotherm measurements were carried out using micromeritic Triflex instruments at IMAP (Paris). Nitrogen sorption data at 77 K were collected on a Micromeritics Tristar-II Plus instrument. The CO_2_ isotherms at 298 K were recorded on a Micromeritics Triflex instrument. In all the cases, the measurements were recorded using ultrahigh purity gases (≥4.8 grade). Before isotherms measurement, the samples were degassed in one step using a Micromeritics SmartVacPrep degas unit: evacuation at 423 K on the degas port (*p* = 10^–6^ mbar), at which point the outgas rate was ≤2 μbar/min.

Parallel to the work at UMONS, a volumetric apparatus (BELSORP Max) was used to measure CO_2_ isotherms with 0.2 g of the sample. The pretreatment conditions were similar to those of the gravimetric measurements. Details about the installation can be found in these publications.^23–25^

### Molecular Simulations

Parallelly, Grand canonical Monte Carlo (GCMC) simulations were carried out to obtain single-component adsorption isotherms of CO_2_ and N_2_. The interactions between the adsorbent and the gases were modeled using the Lennard-Jones (LJ) potential and Coulombic terms. The universal force field (UFF) was used to describe the LJ potentials. The adsorption isotherms were obtained for 298, 308, and 318 K. More details about the procedure for molecular simulations are provided in the previous publication.^19^

### Binary Breakthrough Experiments

Binary breakthrough experiments with two different carrier gases were carried out to establish the mass transfer mechanism. The breakthrough column was 50 cm in height and 2.54 cm in inner diameter, surrounded by a jacket for thermostatting the column with a water bath: in this study, at 303.15 K. There were 3 thermocouples inside the column to record the temperature. The column was packed with about 122 g of MIL-91­(Ti) adsorbent pelletized with the PVB binder. Breakthrough experiments were carried out with 15% CO_2_ and helium and N_2_ as carrier gases. The total flow rate in the experiments was 1 Nl/min. The gas mixture was analyzed continuously by a mass spectrometer [MS] (InProcess Instruments, GAM 200 with a mass range from 1 to 200 amu), calibrated before each set of measurements. The experimental breakthrough curves were then used to calculate the kinetic constants by using a breakthrough curve simulator.

The simulation model for the breakthrough experiments is based on a packed bed system with the MIL-91­(Ti) adsorbent shaped in the form of beads and consists of adsorption rate, mass, energy, and momentum balance equations. The model is developed based on the following assumptions:

1. The ideal gas law is valid.
2. No radial concentration and temperature gradients.
3. Uniformity in adsorbent properties throughout the column.
4. The adsorption rate equation is based on a linear driving force approximation.

The model equations are provided in the Supporting Information. The equations were first converted to a dimensionless form and discretized in the spatial domain by the finite volume method, which resulted in a set of differential algebraic equations (DAEs). The set of DAEs was solved using the ode15s solver in MATLAB. Thirty finite volumes were used, and the Van-leer flux limiter function for avoiding oscillations typical of highly nonlinear systems, such as CO_2_ adsorption.

The fitting of the breakthrough experiments was carried out by regressing the difference between the simulated and experimental concentration and temperature curves. The fitting parameters were the linear driving force coefficient and the internal and external heat transfer coefficient values. The breakthrough experiments were complemented by independent mercury intrusion experiments, which provided values of the porosity, pore size, and particle density.

### Process Simulation

The schematic of the cycle used in this study is shown in Figure 1. This is a 6-step vacuum swing adsorption process^26,27^ and consists of the following steps:

1. Adsorption step: feed enters the column at high pressure, *P*_H_. CO_2_ gets preferentially adsorbed, and N_2_ is collected in the raffinate product.
2. Heavy reflux: The CO_2_ content in the column is enriched by the product coming out of the light reflux step. This stream contains a CO_2_ concentration higher than that of the feed. The column is still at high pressure, *P*_H_.
3. Cocurrent evacuation: the column is evacuated to an intermediate vacuum pressure *P*_INT_ to remove the nitrogen, thereby enabling the cycle to achieve high purity in the subsequent counter-current evacuation step.
4. Counter-current evacuation: the product end is closed, and the column is evacuated from the feed end to remove the CO_2_ product at low pressure *P*_L_.
5. Light reflux: In the light reflux step, the column inlet pressure is maintained at vacuum pressure *P*_L_ and simultaneously purged with the nitrogen product of the adsorption step. The product of this step is completely recycled back to the column to perform the heavy reflux step. The duration of the light reflux and heavy reflux steps is the same.
6. Light product pressurization: The remaining N_2_ from the adsorption step is used to pressurize the column from the product end.

**Figure 1:**
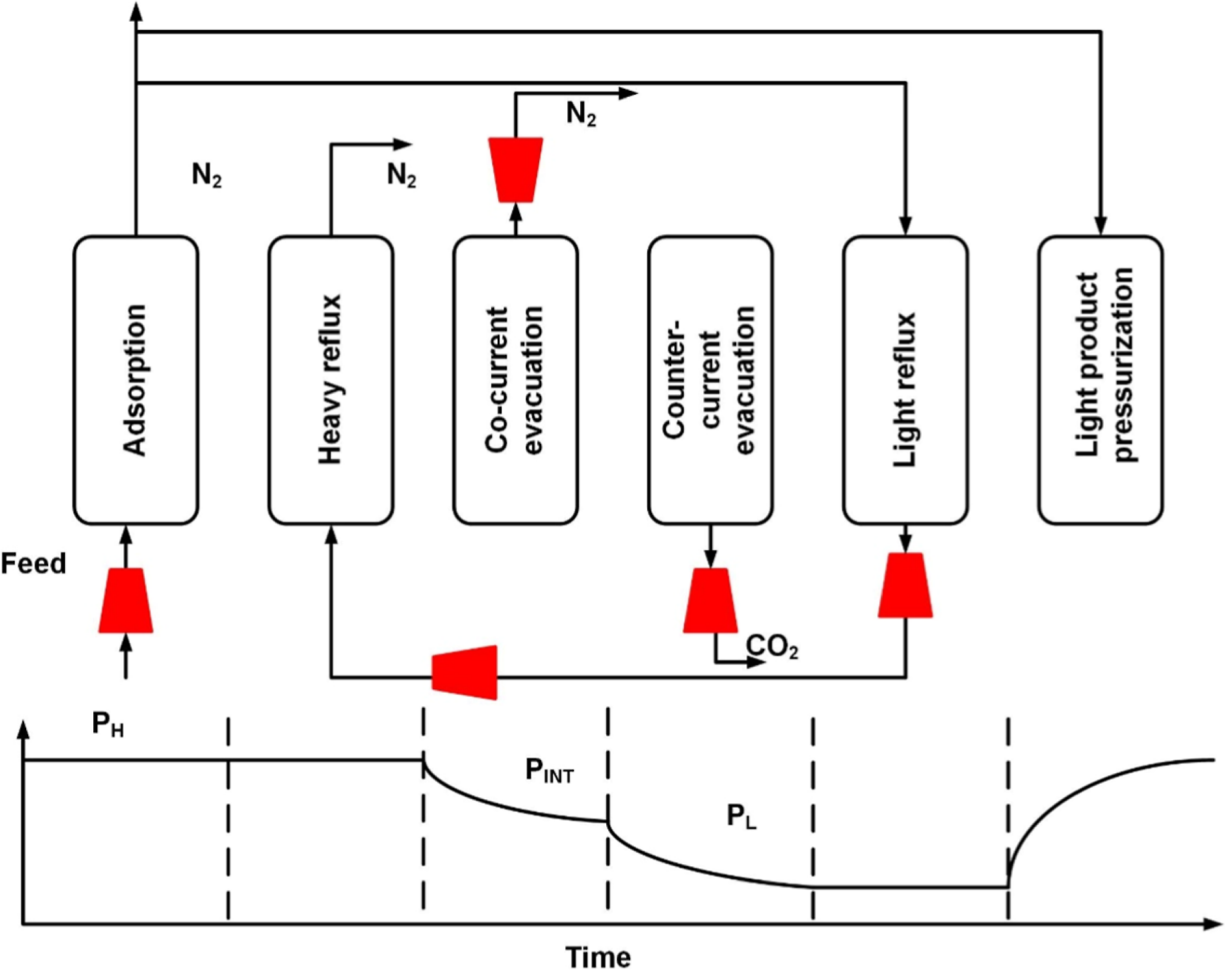
Schematic of the 6-step VPSA process.

The 6-step VPSA process was simulated in MATLAB with the adsorption process model equations mentioned in the previous section, with appropriate boundary conditions for the different steps. These boundary conditions are available in the Supporting Information. The equilibrium and kinetic parameters obtained from the isotherm measurements and breakthrough curves were used as inputs. The VPSA process was simulated under cyclic steady state (CSS) conditions. For the CSS conditions, the mass balance error for five consecutive cycles had to be less than 0.5%.

The performance of the MIL-91­(Ti) in the VPSA cycle is quantified by the following four performance indicators, namely

1 $$CO_{2}\;purity=\frac{Moles_{CO_{2},Cn‐evac}}{Moles_{Total,Cn‐evac}}\times 100$$

2 $$CO_{2}\;recovery=\frac{Moles_{CO_{2},Cn‐evac}}{Moles_{CO_{2},Ads}}\times 100$$

3 $$Specific\;energy=\frac{Energy_{vacuum}+Energy_{compression}}{Moles_{CO_{2},Counter‐currentevacuation}}$$

4 $$Productivity=\frac{Moles_{CO_{2},Cn‐evac}}{V_{ads}\times t_{cycle}}$$

The energy consumed by the vacuum pump and compressors is shown in eqs 5 and 6.

5 $$Energy_{vacuum}=\varepsilon \pi r_{i}^{2}{\frac{\gamma }{\gamma -1}}_{0}\int_{t=0}^{t=t_{vacuum}}vP\left[\frac{1}{\eta \left(P{\left(t\right)}_{vacuum}\right)}{\left(\frac{P_{atm}}{P{\left(t\right)}_{vacuum}}\right)}^{\gamma /\gamma -1}-1\right]dt$$

6 $$Energy_{compress}=\frac{1}{\eta }\varepsilon \pi r_{i}^{2}{\frac{\gamma }{\gamma -1}}_{0}\int_{t=0}^{t=t_{LR}}vP\left[{\left(\frac{\mathrm{P̅}{\left(t\right)}_{in}}{P_{atm}}\right)}^{\gamma /\gamma -1}-1\right]dt$$

For the cocurrent and counter-current evacuation steps, a constant flow vacuum pump is assumed. In the simulations, the pressure downstream of the column is specified, and the pressure at the column exit is calculated from the flow rate to the vacuum pump and the downstream vacuum pressure. Details of the model equations are provided in the Supporting Information, and the dimensionless groups and the boundary conditions are provided in Tables S1 and S2.

The performance indicators are dependent on the following decision variables: adsorption and reflux step durations, the vacuum pressures, the pump flow rates, and the feed flow rate during the adsorption step. A parametric study can only reveal the effect of the different variables on the process performance but cannot identify the optimum of the process performance. This can only be achieved through a detailed optimization study. The purpose of the optimization was to identify minimum energy and maximum productivity values based on purity and recovery targets of >95% and 90%, respectively. In this work, the optimization of the 6-step VPSA process was carried out using a genetic algorithm in MATLAB. The use of genetic algorithm for VPSA process optimization is well investigated in literature,^21,25,26,28–32^ and this work also adopts a similar approach. In total, 4200 simulations were carried out, and the performance of the adsorbent was studied by obtaining Pareto plots of the specific energy consumption and the productivity. More details about the bounds of the decision variables and the other input parameters are provided in the Supporting Information.

## Results and Discussion

### Synthesis and Shaping

#### Physicochemical Characterisations

Figure 2 shows the powder and shaped PXRD images of the MIL-91­(Ti) sample along with the infrared spectrum. The XRD shows a pure powder phase of the adsorbent. The X-ray diffraction pattern of the shaped MOF matches well with that of the powdered form. The infrared spectrum shows the retention of the structural integrity upon granulation of the MOF. The shapes of the CO_2_ adsorption isotherms are similar in powder and shaped forms. When the CO_2_ pressure increases, the CO_2_ uptake decreases on the shaped pellets due to a slight reduction of surface area (on the order of 13%) after the shaping, as seen from Figure 3. The BET area of the shaped sorbent was 350 m^2^/g. This was around 12% lower than that of the precursor powder (460 m^2^/g).

**Figure 2:**
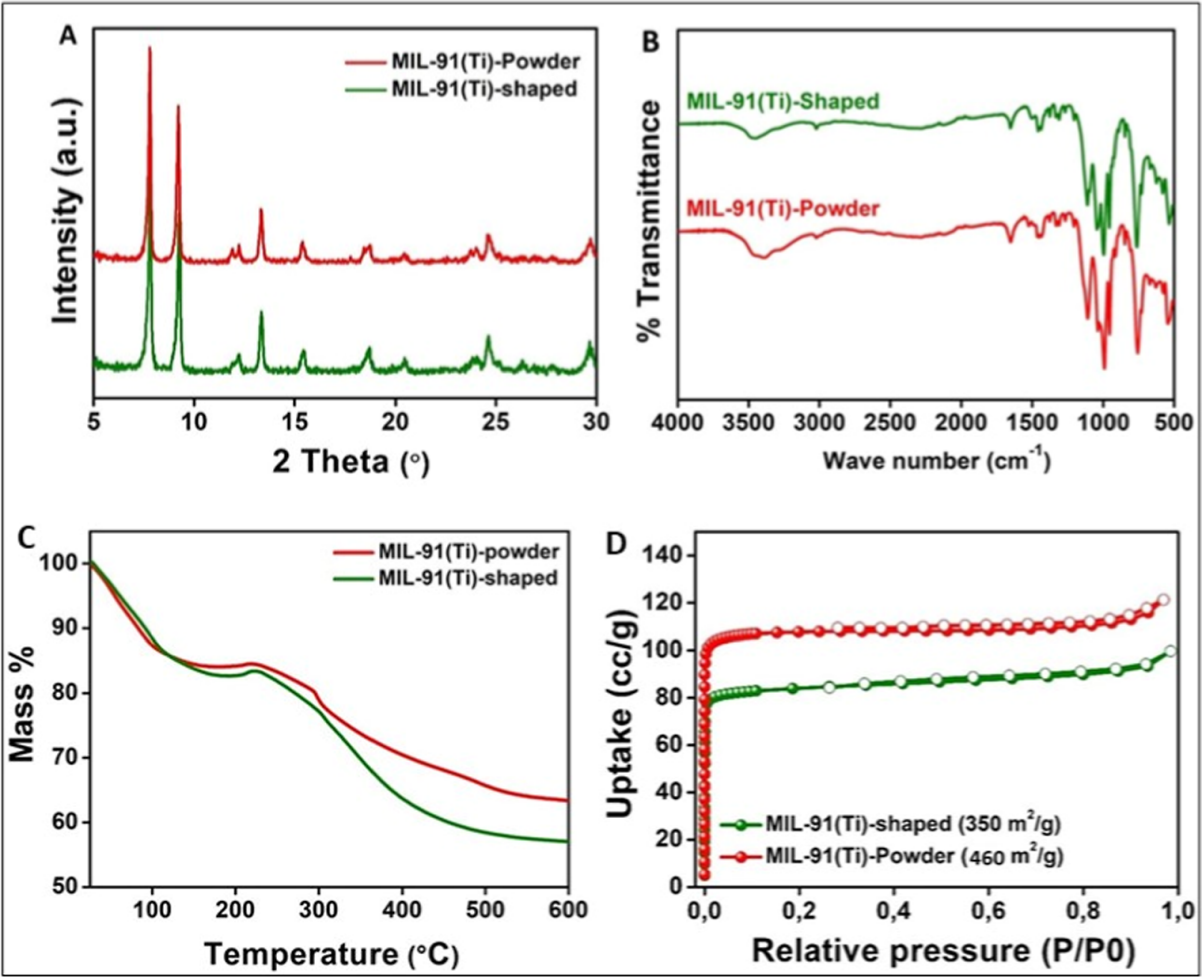
(a) Comparison of PXRD of the MIL-91­(Ti) powder and the shaped (with 3% PVB) form. (b) Infrared spectra of MIL-91­(Ti) shaped with 3% PVB compared to the powder MOF. (c) Thermogravimetric analysis data for MIL-91­(Ti) shaped with 3% PVB compared to the powder MOF. (d) N_2_ adsorption isotherm at 77K and BET data for the powder MOF and its shaped form.

**Figure 3:**
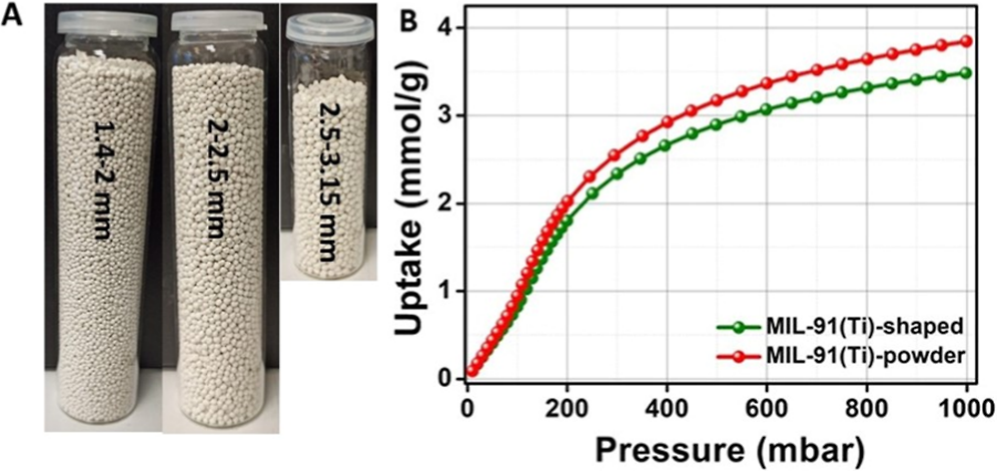
(a) Photographic images of MIL-91­(Ti) shaped with 3% PVB. (b) CO_2_ adsorption isotherm at 298 K of the MIL-91­(Ti) shaped with 3% PVB compared to the powder MOF.

#### Crushing Strength

The average crushing strengths of the different size fractions are shown here in Table 1. The table reports the average crushing strength of three measurements. The values are lower compared to commercial adsorbents shown in the literature,^25^ but in a similar range for MOFs.^34^

**Table 1:** Crushing Strength Values of Different Beads

S. No	size range of the beads (mm)	crushing strength (N)
1	1.4–2.0	6.8
2	2.0–2.5	7.04
3	2.5–3.15	10.27

### Adsorption Isotherms

CO_2_ and N_2_ adsorption isotherms measured in the pelletized sample are shown in Figure 4. CO_2_ adsorbs strongly compared to N_2_, and the adsorption capacity at representative 15–85 flue gas conditions is 1.5 mol/kg for CO_2_ and 0.076 mol/kg for N_2_ at 293 K. The adsorption capacities of some of the commercial sorbents identified as promising MOFs are shown in Table 2. The adsorption capacity of CO_2_ is comparatively less when compared to other sorbents; however, the sorbent also adsorbs considerably less nitrogen. This may be an advantage in a VPSA process to help achieve high CO_2_ purities. The CO_2_ adsorption isotherms measured with the gravimetric and the volumetric system agree with one another, as seen from Figure 4c. Observing carefully, the CO_2_ adsorption isotherm seems to exhibit an inflection point and is more predominant in the volumetric isotherm at 293 K and in the 313 K isotherm. The pressure at which the inflection point occurs increases as a function of temperature. Isotherms were also measured at 343 and 373 K by the volumetric apparatus, and these isotherms did not show any inflection below 1 bar pressure (Figure S2 in the SI). This suggests that there could be a possibility of a mild breathing effect when CO_2_ adsorbs onto the MIL-91 surface. Some MOFs may exhibit breathing phenomena that modify the pore size during adsorption, leading to type IV isotherms. Such a phenomenon has been widely reported in literature for CO_2_ adsorption in certain metal organic frameworks.^42–44^

**Figure 4:**
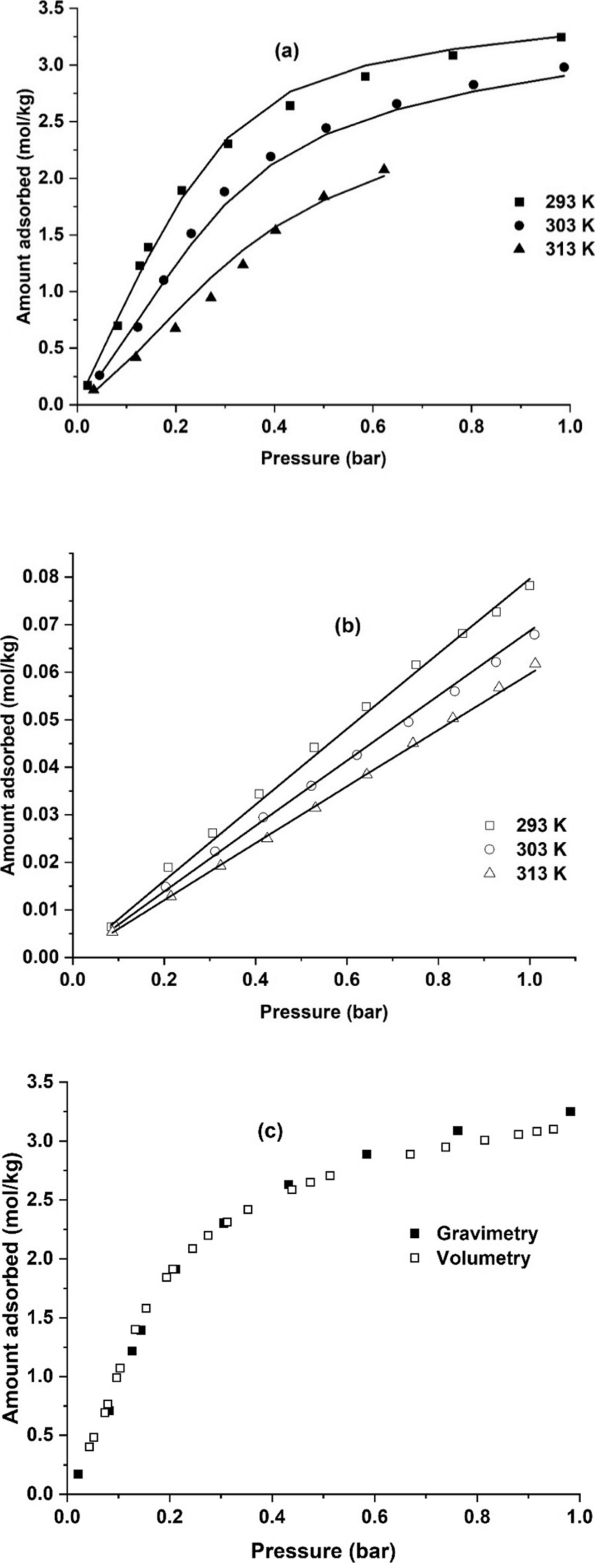
(a) CO_2_ and (b) N_2_ adsorption isotherms on MIL-91 Ti pellets with 3% PVB binder and (c) comparison of isotherms measured by volumetry and gravimetry. Lines denote the dual site Langmuir model with the distribution function.

**Table 2:** Single Component CO_2_ and N_2_ Adsorption Capacities at 298 K for Selected Sorbents

adsorbent	CO_2_ capacity, 0.15 bar (mol/kg)	N_2_ capacity, 0.85 bar (mol/kg)	reference
MIL-91 (Ti)	1.2	0.06	this work
CALF-20	2.4	0.25	^12^
CPO-27-Ni	4.97	0.97	^35^
UTSA-16	2.25	0.12	^36^
Zeolite 13X	3.63	0.5	^37^
MIL 120	2.25	0.33	^38^
MIL-96	1.4	0.37	^39,40^
activated carbon	1	0.35	^41^

In Figure 5, the adsorption isotherms generated from molecular simulations are shown. It is worth noting that the isotherms generated from molecular simulations are based on a crystalline material. A real adsorbent, however, considers the presence of binders, and the sorbent is shaped in the form of a pellet. As the shaped MOF comprises 3% PVB, the isotherms from the molecular simulations were corrected by 3%. The resultant capacities at 0.15 bar of CO_2_ and 0.85 bar of nitrogen were 1.78 mol/kg and 0.2 mol/kg. Furthermore, one can also see that the isotherms generated by the molecular simulations did not capture the inflection point, which was observed in the experiments for CO_2_. This could be a result of the generic universal force field (UFF), which was used to estimate the adsorption isotherms of the compound CO_2_. Figure 5c captures the difference in shape between the experimental and the simulated isotherms. The simulated isotherms appear steeper compared to the experimental isotherms. The saturation capacity is lower for the simulated isotherms compared to the experimental isotherms.

**Figure 5:**
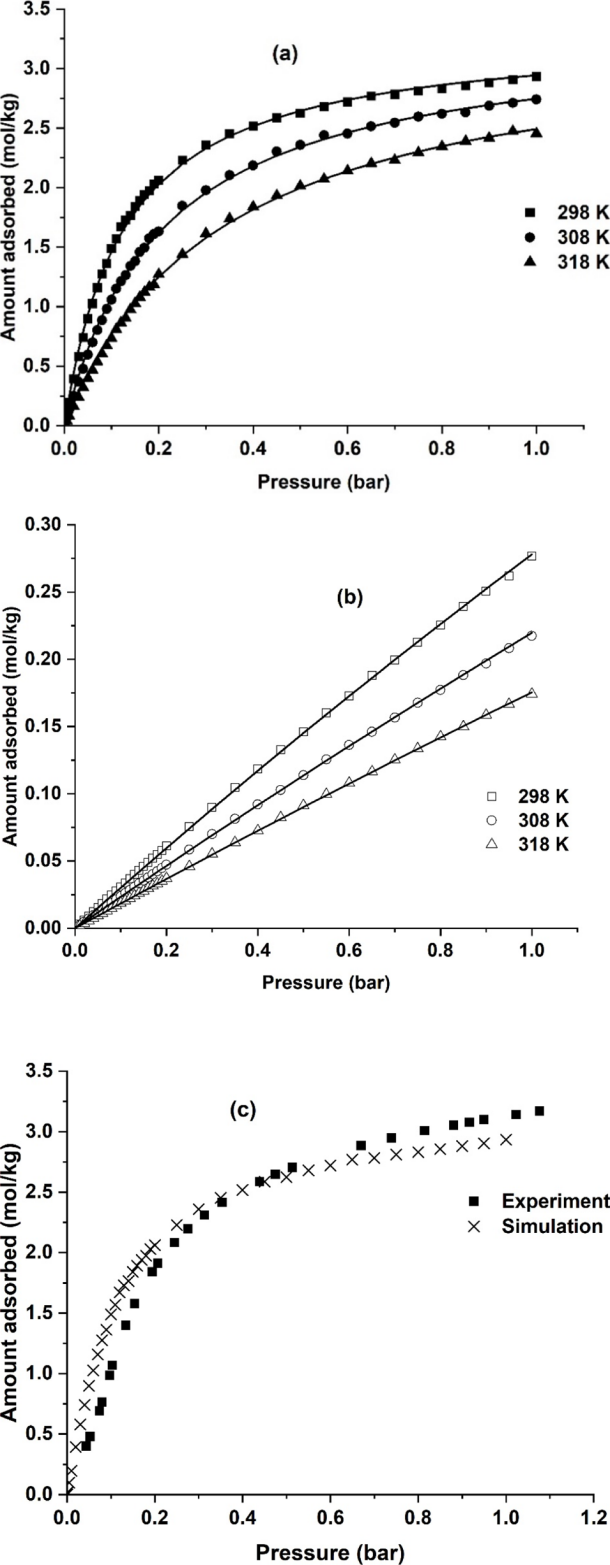
(a) CO_2_ and (b) N_2_ isotherms generated by molecular simulations. Figure (c) compares the experimental isotherms at 293 K and simulated isotherms at 298 K. Lines denote Langmuir model fits.

The experimental CO_2_ adsorption data were fitted to the following model by eq 7. The model is a dual-site Langmuir model (DSL) with a distribution function between small and large pores depending on the concentration of the gas phase.^43^

7 $$q^{*}=\left(1-\varphi \right)\frac{q_{s1}{b_{01}e}^{-\Delta U_{1}/RT}c}{1+{b_{01}e}^{-\Delta U_{1}/RT}c}+\left(\varphi \right)\frac{q_{s2}{b_{02}e}^{-\Delta U_{2}/RT}c}{1+{b_{02}e}^{-\Delta U_{2}/RT}c}$$

The distribution function φ is defined as

8 $$\varphi =0.5\left(1+\mathrm{erf}\left(\frac{c-m}{s}\right)\right)$$

Here, *m* and *s* are defined as the mean and the standard deviation of the Gaussian function.

The nitrogen adsorption was defined by a DSL model

9 $$q^{*}=\frac{q_{s1}{b_{01}e}^{-\Delta U_{1}/RT}c}{1+{b_{01}e}^{-\Delta U_{1}/RT}c}+\frac{q_{s2}{b_{02}e}^{-\Delta U_{2}/RT}c}{1+{b_{02}e}^{-\Delta U_{2}/RT}c}$$

The saturation capacities were kept the same for nitrogen for thermodynamic consistency, and the b0 and the Δ*U* parameters were the same for the two sites owing to the linear shape of the isotherm. The isotherms generated by the molecular simulations were fitted to a single site Langmuir model, which was sufficient to describe the adsorption of both CO_2_ and N_2_.

The fitting of the isotherm was carried out by minimizing the error between the experimental data and the model. The parameters, the residual, along with selectivity and isotherm nonlinearity are shown in Tables 3 and 4, and the model fits are described as lines in Figures 4 and 5. The CO_2_ isotherm was described well by the DSL model with the distribution function. Table 3 shows that the deviation for the parameters in the first site is bigger; however, site 1 contributes to <10% of the overall equilibrium capacity. The limiting selectivity, which is the ratio of the Henry’s constants, is provided in Table 3, and MIL-91 has a high selectivity of around 276. The isotherm nonlinearity is defined as the ratio of the equilibrium and the saturation capacities. The higher the value is, the steeper the isotherm is. MIL-91 has a nonlinearity of 0.17, when one compares the values at 298 K and 0.15 bar, which is indicative of a less steep isotherm. The steepness of the isotherm determines the evacuation pressure in the VPSA process, which affects the overall energy consumption.

**Table 3:** Adsorption Isotherm Parameters for the Pellet

parameter	experiment	
	CO_2_	N_2_
*q*_s1_ (mol/kg)	2.89 ± 1.3	2.89 ± 1.3
*b*_01_ (m^3^/mol)	2.415 × 10^–5^ ± 7.22 × 10^–5^	8.7 × 10^–6^ ± 2 × 10^–6^
Δ*U*_1_ (J/mol)	–16080.4 ± 8600	–8622.2 ± 20
*q*_s2_ (mol/kg)	3.71 ± 0.14	3.71 ± 0.14
*b*_02_ (m^3^/mol)	5.62 × 10^–10^ ± 4 × 10^–10^	8.7 × 10^–6^ ± 2 × 10^–6^
Δ*U*_2_ (J/mol)	–47653.2 ± 6400	–8622.2 ± 20
*m*	3.43	
*s*	6.93	
mean squared error	0.005	3.6 × 10^–5^
Henry’s constant at 298 K	551.1	1.99
limiting selectivity at 298 K	276	
isotherm nonlinearity 0.15 bar 298 K	0.17	
CO_2_ capacity 0.15 bar 298 K (mol/kg)	1.14	
N_2_ capacity 0.85 bar 298 K (mol/kg)	0.063	

**Table 4:** Adsorption Isotherm Parameters for the Simulated Isotherms

parameter	molecular simulations	
	CO_2_	N_2_
*q*_s_ (mol/kg)	3.31 ± 0.01	3.31 ± 0.01
*b*_0_ (m^3^/mol)	1.38 × 10^–7^ ± 2.1 × 10^–8^	2.5 × 10^–6^ ± 7.7 × 10^–8^
Δ*U* (J/mol)	–35107.6 ± 382	–16908.7 ± 77.5
mean squared error	0.007	7 × 10^–7^
Henry’s constant at 298 K	470.9	5.48
limiting selectivity at 298 K	85.9	
isotherm nonlinearity 0.15 bar 298 K	0.53	
CO_2_ capacity 0.15 bar 298 K (mol/kg)	1.84	
N_2_ capacity 0.85 bar 298 K (mol/kg)	0.202	

As mentioned earlier, the molecular simulations predicted stronger adsorption for the CO_2_ and N_2_ in MIL-91. In line with the capacity numbers, the values of the Henry’s constant and the limiting selectivity are different for the actual data and the simulated isotherms, as shown in Table 4. Due to a strong nitrogen adsorption predicted by the molecular simulations, a lower selectivity value is obtained (85.9 vs 276). The isotherm obtained by the molecular simulation had a higher nonlinearity, which is a consequence of the high CO_2_ capacity and the Langmuir isotherm. The effect of such differences on the overall performance will be seen in the process modeling in the subsequent sections. Tables S3 and S4 contain the isotherm data obtained from experiments and molecular simulations.

### Mercury Intrusion Data

The results from the mercury intrusion experiments are listed in Figure 6. Two peaks were observed, one corresponding to a macropore of 160 nm and a second peak with a mesopore size of 3 nm. The average porosity was 37.8%, and the particle density was 1075.4 kg/m^3^. The results from the mercury intrusion are summarized in Table 5. The mercury intrusion data, coupled with the volumetric experiments, were, used to analyze the breakthrough experiments and used in the process modeling.

**Figure 6:**
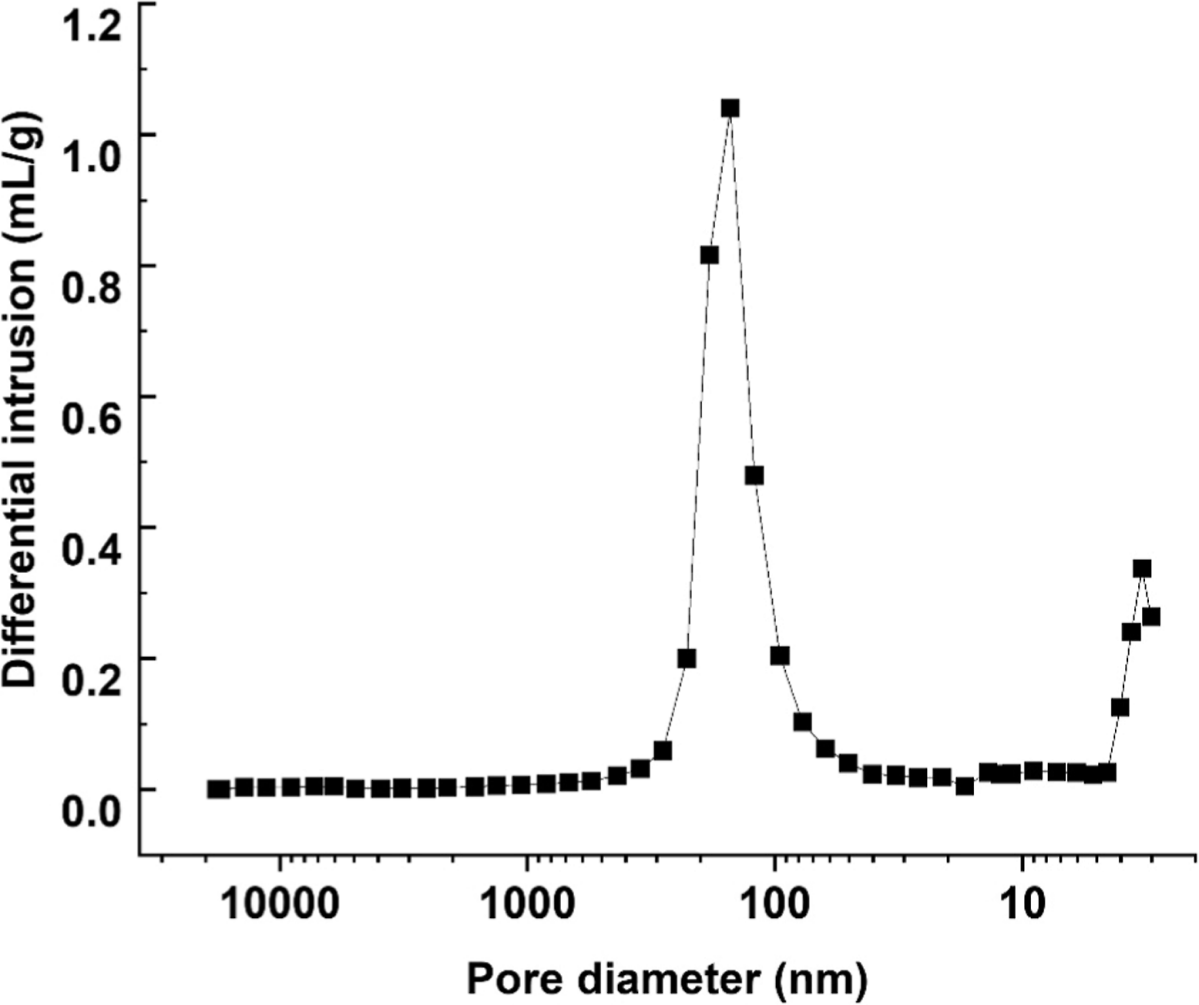
Mercury intrusion curve in the MIL-91­(Ti) beads.

**Table 5:** Summary of the Mercury Intrusion Experiments

parameter	value
macropore size (nm)	159
porosity	0.378
particle density (kg/m^3^)	1075.4
skeletal density (kg/m^3^)	1726.5

### Binary Breakthrough Experiments

A pelletized adsorbent consists of microporous adsorbent crystals held together by a binder. The adsorption rate of the gas is governed by the diffusion through the external film, macropores, and micropores. The film resistance is usually negligible. The diffusion through the macropores is a function of the carrier gas or the pellet size, whereas the micropore diffusion process is independent of both the pellet size and the carrier gas.

To establish whether the adsorption of CO_2_ was governed by macropores or micropores, two different experiments were carried out with helium and nitrogen as carrier gases. The choice of the two carrier gases was based on the significant differences in the binary molecular diffusivity with CO_2_. The molecular diffusivity value for the CO_2_–He pair is about 0.63 cm^2^/s, while the CO_2_–N_2_ binary diffusivity is around 0.16 cm^2^/s at 298 K. The mole fraction of CO_2_ was fixed at 0.15 for both experiments. In Figure 7, the experimental breakthrough responses are plotted for the two different carrier gases. A plot of the normalized time vs CO_2_ mole fraction shows a difference in the shape of the breakthrough curve. The breakthrough curve with N_2_ appears to have an earlier breakthrough compared to that of the curve with He as the carrier gas. The two curves cross at a CO_2_ mole fraction of 0.075, and the breakthrough with the helium carrier gas reaches the maximum value faster than the curve with the N_2_ carrier gas. This is an indication that the adsorption of CO_2_ in MIL-91­(Ti) is governed by diffusion in the macropores.

**Figure 7:**
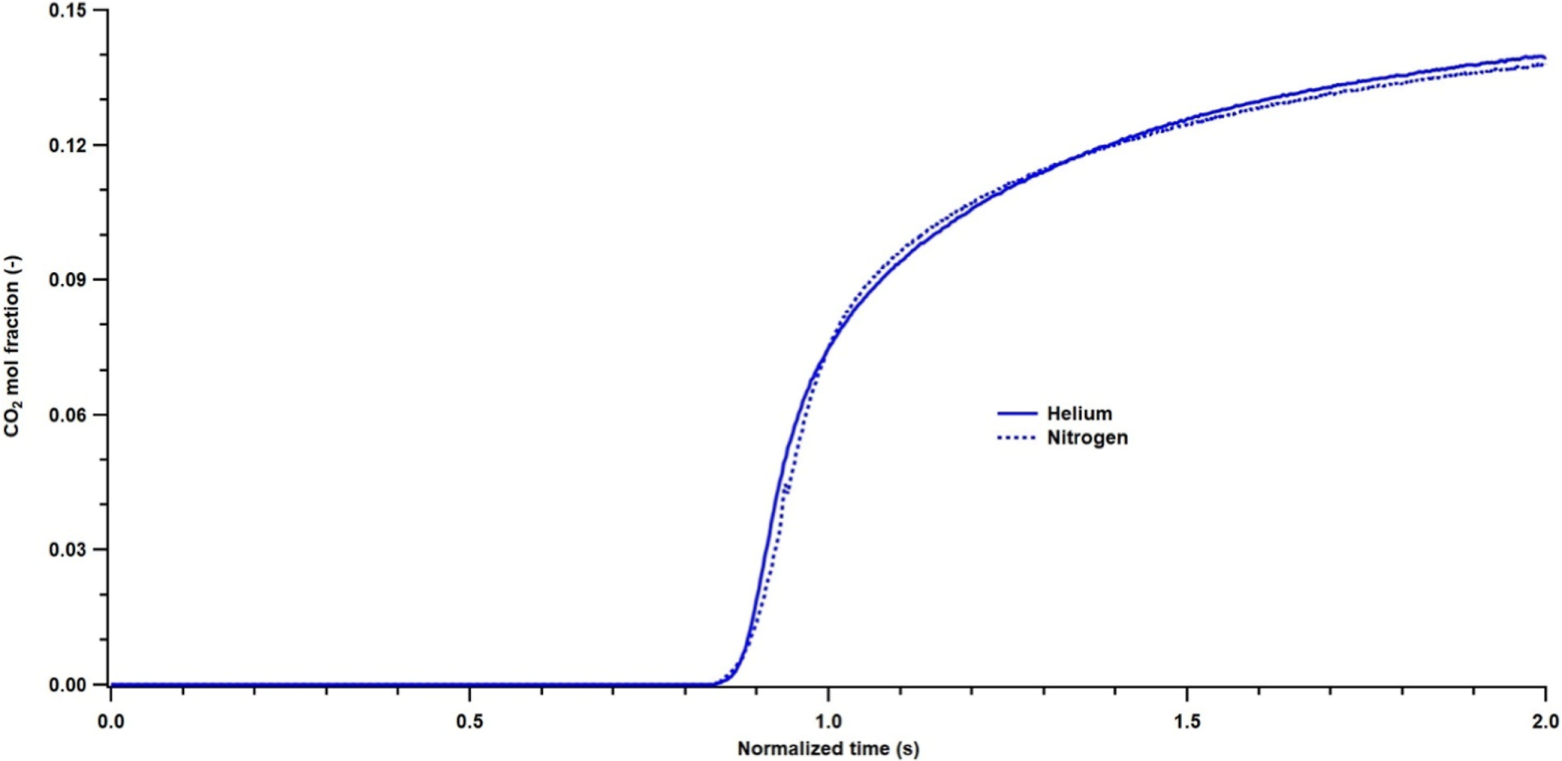
Experimental breakthrough curves with 15% CO_2_ with helium and nitrogen as carrier gases.

The adsorption rate constants for the two experiments were obtained using the adsorption process model described in the previous section. The adsorption breakthrough curve was simulated using the model, and the error between the simulated and experimental breakthrough curves was minimized. The adsorption process model equations are defined in the Supporting Information. The adsorption rate was described by a linear driving force model and contains the adsorption rate constant or the linear driving force coefficient (*k*_LDF_). The heat balance equation accounts for the heat transfer between the fluid to the column wall and the column wall to the external ambient and is defined by internal and external heat transfer coefficients (*h*_i_, *h*_0_). These parameters affect the shape of the concentration and temperature breakthrough curves. Therefore, the decision variables for the fitting exercise were the linear driving force coefficient, heat transfer coefficient values, and the pre-exponential values *b* ^0^ of the modified dual site Langmuir isotherm. First, the helium experiment was fitted, and in the next step, the fitting of the nitrogen experiment was carried out with the isotherm parameters obtained from the helium experiment.

The breakthrough experiments are coupled with independent mercury intrusion experiments. From the mercury intrusion experiments, porosity and pore size information were obtained.

For a binary system, the diffusion through macropores is governed by the molecular and Knudsen diffusion in the following manner.^45^

10 $$D_{Macro}=\frac{D_{Mol}D_{K}}{D_{Mol}+D_{K}}$$

The molecular and Knudsen diffusivities are calculated as follows.^46^

11 $$D_{Mol}=\frac{0.001858T^{3/2}}{P\sigma_{1,2}^{2}\Omega }\left(\sqrt{\frac{1}{M_{1}}+\frac{1}{M_{2}}}\right)$$

12 $$D_{Knudsen}=0.6715r_{pore}\sqrt{\frac{T}{M}}$$

Here, the Knudsen diffusivity is corrected by the Derjaguin correction factor.^47^

The linear driving force approximation can be expressed as.

13 $$k_{LDF}=\frac{15\frac{\varepsilon_{p}}{\tau }D_{macro}}{r_{p}^{2}\frac{\partial q}{\partial c}}$$

Applying equations and the fitted linear driving force correlation, one can estimate the tortuosity. Table 6 shows the values of the fitted linear driving force coefficient, and there is good agreement between the values of the tortuosity obtained in the two experiments. Figure 8 shows a comparison of the experimental and simulated breakthrough curves. A good match can be observed between the real and simulated curves. The kinetic constants obtained from the breakthrough curve analysis were then used for the process modeling and optimization to estimate the following process performance indicators, namely, CO_2_ purity, CO_2_ recovery, energy consumption, and productivity.

**Table 6:** Summary of the Breakthrough Experiments

experiment	*D*_M_ (m^2^/s)	*D*_K_ (m^2^/s)	*D*_macro_ (m^2^/s)	*k*_fitted_ (1/s)	d*q*/d*c*	τ
CO_2_–He	6.3 × 10^–5^	2.8 × 10^–5^	1.94 × 10^–5^	0.21	127	2.58
CO_2_–N_2_	1.6 × 10^–5^	2.8 × 10^–5^	1.06 × 10^–5^	0.34	119	2.43

**Figure 8:**
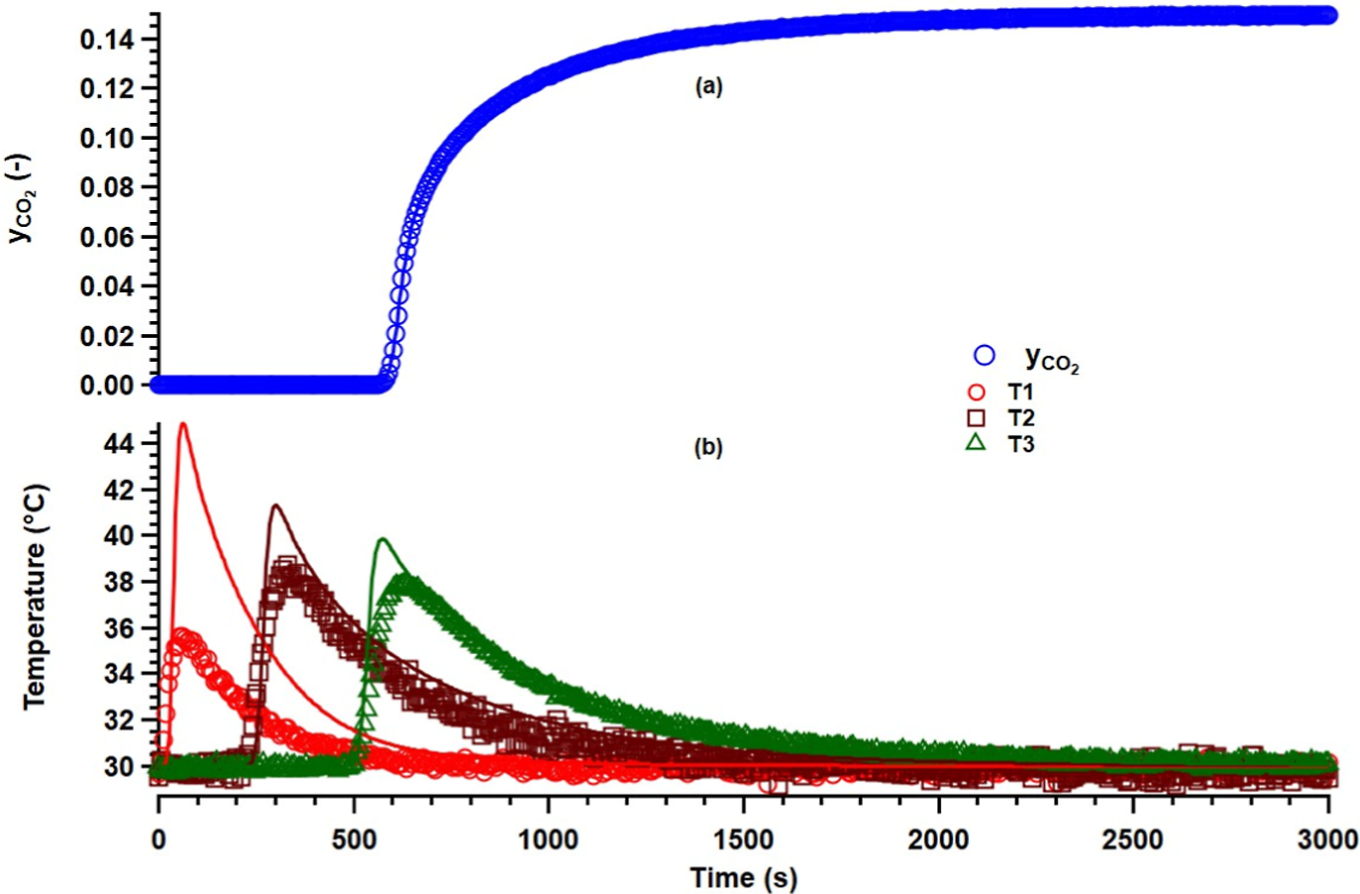
Comparison of experimental and theoretical breakthrough curves. The symbols denote experimental data, while the lines denote the simulated data.

### Process Optimization

The process simulations were performed based on the assumptions of a dry flue gas available at 298 K. Independent stability tests revealed that the water adsorption was strong, and it affected the adsorption of CO_2_. This has already been pointed out in earlier literature.^48^ The presence of water may result in increased CO_2_ losses in the VPSA process, which can impact the recovery. Further, the stability tests showed that the presence of contaminants such as SO_X_ and NO_X_ affected the CO_2_ adsorption. The study aims to evaluate the performance of the adsorbent under dry conditions, and the stability tests are not a part of this work. More details about the stability tests shall be provided in a separate publication. Additionally, the choice of dry flue gas and no contaminants enabled comparison with VPSA process simulations reported in the literature. It is worth reiterating that the goal of this work is to study whether the sorbent can achieve 95% purity and 90% recovery in a 6-step VPSA process, and other indicators like cost numbers are beyond the scope of the current work.

The process optimization was carried out using the information from the adsorbent characterization. Two adsorption isotherms were chosen: (1) the adsorption isotherms measured on the pellets (experimental isotherms) and (2) the adsorption isotherms obtained from molecular simulations and corrected for the 3% binder (simulated isotherms). The length and the diameter of the column were kept similar to published literature.^23,25,32–34^ The feed temperature was assumed to be 298 K. Select input parameters are provided in Table S5 of the Supporting Information. The competition between CO_2_ and N_2_ was described as follows for the experimental data

14 $$q_{{CO}_{2}}^{*}=\left(1-\varphi \right)\frac{q_{s1}{b_{01,{CO}_{2}}e}^{-\Delta U_{1,{CO}_{2}}/RT}c_{{CO}_{2}}}{1+{b_{01,{CO}_{2}}e}^{-\Delta U_{1,{CO}_{2}}/RT}c_{{CO}_{2}}+{b_{01,N_{2}}e}^{-\Delta U_{1,N_{2}}/RT}c_{N_{2}}}+\left(\varphi \right)\frac{q_{s2}{b_{02,{CO}_{2}}e}^{-\Delta U_{2,{CO}_{2}}/RT}c_{{CO}_{2}}}{1+{b_{02,{CO}_{2}}e}^{-\Delta U_{2}/RT}c_{{CO}_{2}}{{+b}_{02,N_{2}}e}^{-\Delta U_{2,N_{2}}/RT}c_{N_{2}}}$$

15 $$q_{N_{2}}^{*}=\frac{q_{s1}{b_{01,N_{2}}e}^{-\Delta U_{1,N_{2}}/RT}c_{N_{2}}}{1+{b_{01,{CO}_{2}}e}^{-\Delta U_{1,{CO}_{2}}/RT}c_{{CO}_{2}}+{b_{01,N_{2}}e}^{-\Delta U_{1,N_{2}}/RT}c_{N_{2}}}+\frac{q_{s2}{b_{02,N_{2}}e}^{-\Delta U_{2,N_{2}}/RT}c_{N_{2}}}{1+{b_{02,{CO}_{2}}e}^{-\Delta U_{2}/RT}c_{{CO}_{2}}{{+b}_{02,N_{2}}e}^{-\Delta U_{2,N_{2}}/RT}c_{N_{2}}}$$

Similarly, for the MS data, a competitive single site Langmuir model was used.

4200 simulations were carried out in total for each of the cases. Then the points satisfying 95% purity and 90% recovery were taken out, and a Pareto plot was obtained for the energy and productivity values (Figure 9). Figure 9 plots the results from both the experimental and the simulated isotherms. It is important to highlight here that the purity values for the simulated isotherms were around 94%, while the recovery values were between 87.5% and 89.6%. The minimum energy for the experimental adsorption isotherms was 0.88 MJ/kg, and the maximum productivity was 0.76 mol/m^3^ ads s. For the simulated adsorption isotherms, the values were 1.67 MJ/kg and 0.39 mol/m^3^ ads s. The operating conditions and the cyclic steady state profiles are provided in the Supporting Information for the minimum energy conditions (Table S6 and Figures S3 and S4).

**Figure 9:**
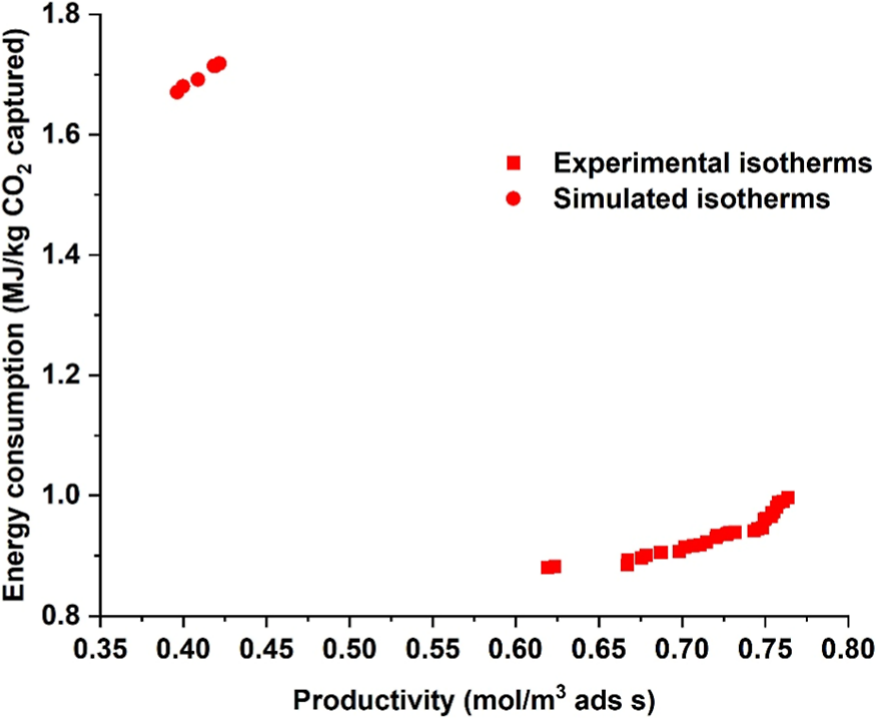
Productivity vs energy Pareto curves.

Figure 10 shows a plot of the volume of flue gas entering the cycle vs productivity. The optimizer chose adsorption step durations of 22–23 s for the simulated adsorption isotherms and 48–52 s for the experimental adsorption isotherms. Due to a higher capacity predicted by the molecular simulations, the feed velocities were higher (1.4–1.5 m/s) in this case compared to the experimental adsorption isotherms (1.15–1.28 m/s). Nevertheless, the volume of flue gas/CO_2_ entering the column during the adsorption step is higher for the experimental adsorption isotherms due to the longer adsorption step duration. This is why the productivity is higher for the simulations carried out with the experimental adsorption isotherms.

**Figure 10:**
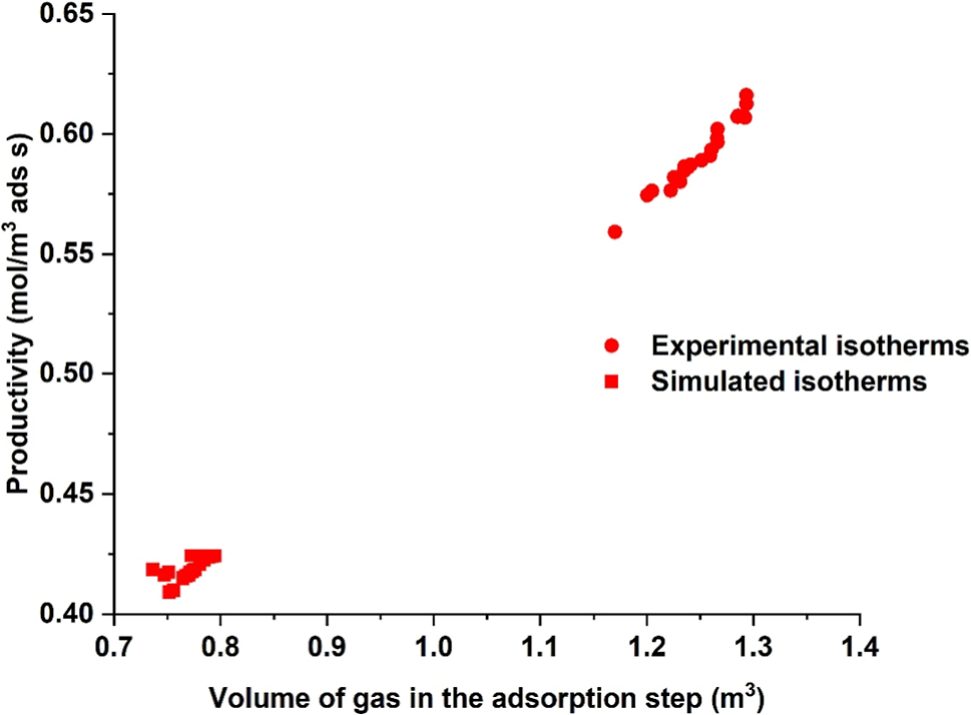
Productivity as a function of the volume of flue gas entering the adsorption step.

In Figure 11, one can see a plot of the evacuation pressures vs the energy consumption. From the previous section on the adsorption isotherms, it has been established that the molecular simulations predict higher CO_2_ and N_2_ capacities. Further, the simulated CO_2_ adsorption isotherms were steeper compared to the experimental adsorption isotherms, and this can be seen from the higher nonlinearity value. The stronger nitrogen adsorption meant that the cocurrent evacuation step required a deeper vacuum of 0.23 bar as opposed to a moderate 0.43 bar for the experimental isotherms. Similarly, for the counter-current evacuation steps, the values of the evacuation pressures were around 0.13 bar as opposed to 0.17 bar observed for the experimental adsorption isotherms. Due to a deeper vacuum, the flow rates to the vacuum pumps were higher for the simulated isotherms case than the actual isotherms on pellets. The deeper vacuum meant that the energy consumption was higher for the simulated adsorption isotherms. The strong nitrogen adsorption meant that the predicted isotherms were able to achieve nearly and not above 95% purity.

**Figure 11:**
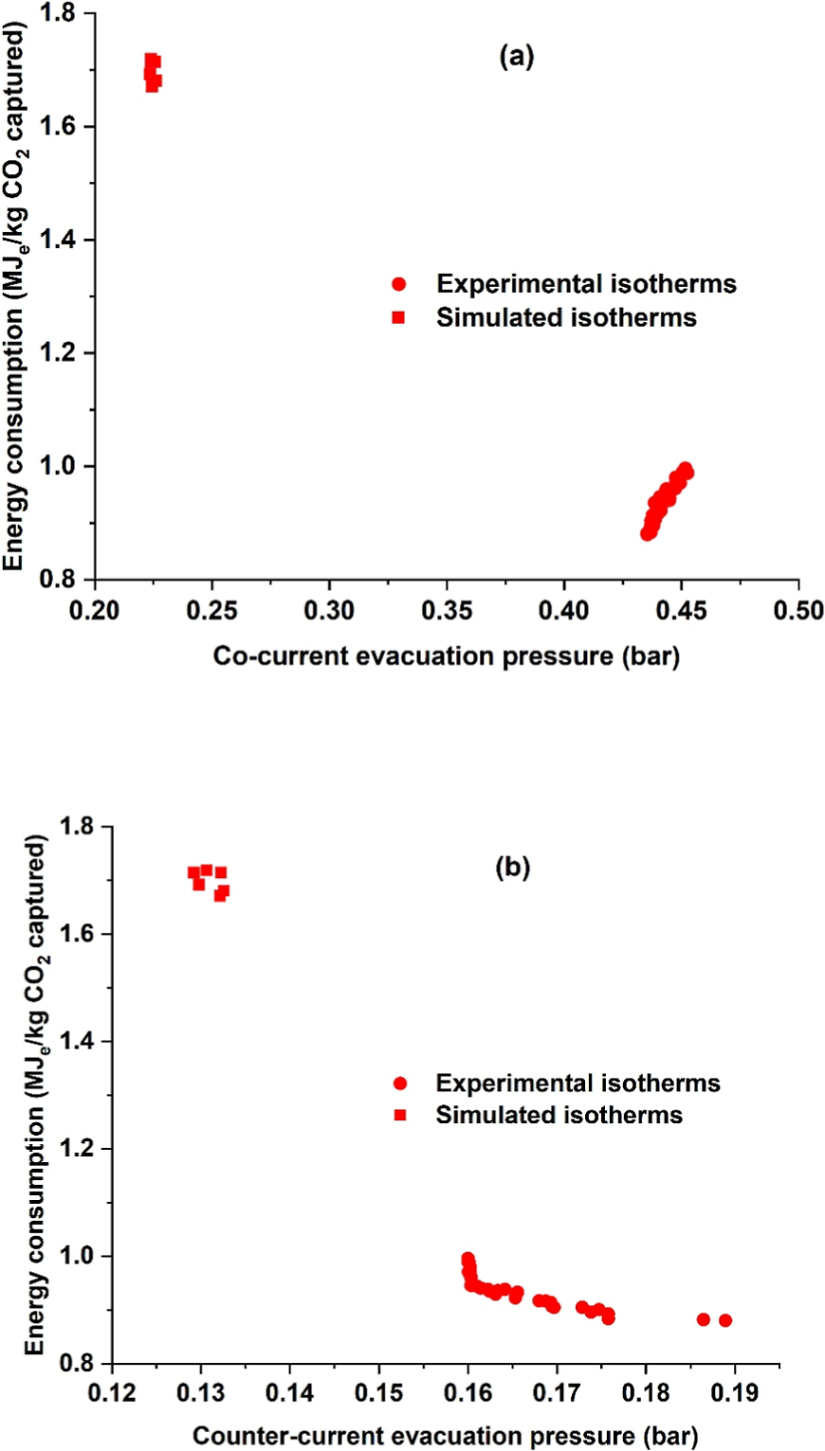
Energy consumption as a function of (a) cocurrent evacuation pressure and (b) counter-current evacuation pressure.

In Table 7, a comparison of the performance of the different sorbents are provided. The differences in performance may not only be due to the adsorption capacities but may also be due to the differences in the choice of operating conditions as well as the calculation of the energy consumption of the vacuum pump. The zeolite 13X and the UTSA-16 MOF have considerably higher capacity values and hence the performance was significantly better. Furthermore, in a couple of studies, the evacuation step was modeled as an exponential decay in pressure. In the remaining studies, including the current work, a constant flow vacuum pump with efficiencies varying with pressure was considered.

**Table 7:** Comparison of the Energy Productivity Values in a 6-Step VPSA Process for Selected Sorbents Shaped in the Form of a Pellet

adsorbent	minimum energy (MJ/kg)	maximum productivity (mol/m^3^ ads s)	reference
MIL-91(Ti)	1.03	0.61	this work
HKUST-1	0.92	0.78	^49^
amino silane	1.2	0.14	^25^
Zeolite 13X	0.58	4.7	^26^
Zeolite 13X	1.18	0.83	^27^
UTSA-16	0.47	4.28	^26^

MIL-91­(Ti) performs reasonably well and was able to meet the desired purity and recovery targets. The performance of the CPO-27-Ni MOF was also studied in parallel. This adsorbent has a significantly higher CO_2_ capacity compared to the MIL-91 (Ti) MOF. However, the MOF was not able to achieve the desired purity targets due to the high nitrogen affinity in this adsorbent. This further reiterates the importance of N_2_ adsorption and shows that MIL-91­(Ti) is one of the promising MOFs for CO_2_ capture from a feed containing 15% or more CO_2_.

## Conclusions

In this work, the microporous Ti bisphosphonate MIL-91­(Ti) was synthesized, scaled-up, and shaped prior to being evaluated for the separation of CO_2_ over N_2_ in a flue gas separation process. The adsorbent high CO_2_ working capacity and good CO_2_/N_2_ selectivity were first validated prior to a series of binary breakthrough experiments. Through process simulations, the ability of this sorbent to achieve desired purity and recovery targets of 95% and 90%, respectively, was demonstrated. However, the strong hydrophilic character of the sorbent is a key limitation, and therefore, the flue gas should be dried sufficiently before the CO_2_ capture process. The study also highlights the necessity for more accurate force fields, especially for nitrogen sorption, in order to avoid any overprediction of the capacity as well as the shape of the isotherm. This points out the dependence of the process performance not only on the CO_2_ sorption but also on the accuracy with which the nitrogen adsorption isotherms are estimated. Further work is necessary to estimate the overall cost of the VPSA process.

## Supplementary Material



## Notes

Model equations, boundary conditions, and CSS profiles for the optimization, along with the raw isotherm data (PDF)

## List of symbols and notations, Greek symbols

*b*_0_: affinity coefficient of the dual-site Langmuir isotherm for sites 1 and 2
*c*_i_: gas phase concentration of component i (mol/m^3^)
*D*_macro_: macropore diffusion coefficient (m^2^/s)
*D*_mol_: molecular diffusion coefficient (m^2^/s)
*D*_K_: Knudsen coefficient (m^2^/s)
*h*_i_: internal heat transfer coefficient (W/m^2^ K)
*h*_0_: external heat transfer coefficient (W/m^2^ K)
*k*_LDF_: linear driving force coefficient (s^–1^)
*M*: molecular weight of the gas (kg/mol)
*P*: total pressure in the system (Pa)
*P*_Vacuum_: vacuum pressure in the cocurrent/counter-current evacuation step (Pa)
*P*_ATM_: ambient pressure (Pa)
*q*_i_: solid phase concentration (mol/m^3^)
*q*_s_: solid phase concentration at saturation (mol/m^3^)
*q*_i_*: equilibrium solid phase concentration (mol/m^3^)
*R*: gas constant (J/mol/K)
*r*_i_: Column internal radius (m)
*r*_p_: pellet radius (m)
*r*_pore_: pore radius (cm)
*T*: temperature inside the column (K)
*t*: time (s)
Δ*U*: internal energy of adsorption (J/mol)
*v*: interstitial velocity (m/s)
*y*_i_: mole fraction of component i
ε: bed void fraction
ρ_s_: density of the adsorbent (kg/m^3^)
σ: average collision diameter (Å)
Ω: collision integral
η: vacuum pump efficiency
γ: ratio of the specific heats
μ: viscosity of gas mixture (Pa s)
φ: distribution function for the DSL model
