# Supplementary material for: Evaluating the Performance of a Microporous Ti Bisphosphonate MOF for Postcombustion Carbon Capture by Vacuum Pressure Swing Adsorption
Source: Ind Eng Chem Res. 2025 Jul 23;64(31):15404–16. doi: 10.1021/acs.iecr.5c00734 (PMC12333008; doi:10.1021/acs.iecr.5c00734)
Supplement: Supplementary file 1 [file ie5c00734_si_001.pdf]

Evaluating the performance of a microporous Ti bisphosphonate MOF for post-combustion carbon capture by vacuum pressure swing adsorption (VPSA)

Shreenath Krishnamurthy[a*], Nicolas Heymans[b], Mohammad Wahidduzzaman[c], Guillaume Maurin[c], Shyamapada Nandi[d], Richard Blom[a*], Debanjan Chakraborthy[d], Farid Nouar[d], Christian Serre[d], Giorgia Mondino[a], Georges Mouchaham[d] and Guy De Weireld[b*]

[a] Process Technology, SINTEF INDUSTRY, Oslo, 0373, Norway

[b] Thermodynamics and Mathematical Physics Unit, University of Mons (UMONS), 7000 Mons Belgium

[c] ICGM, University of Montpellier, CNRS, ENSCM, 34293 Montpellier, France

[d] Institut des Matériaux Poreux de Paris, ENS, ESPCI Paris, CNRS, PSL University, Paris 75005 France

Corresponding Author's contacts : Shreenath.Krishnamurthy@sintef.no

Model equations in the dimensionless form

Component mass balance:

$$\frac{\partial y_i}{\partial \tau} = \frac{1}{Pe}\frac{\bar{T}}{\bar{P}}\frac{\partial}{\partial Z}\left(\frac{\bar{P}}{\bar{T}}\frac{\partial y_i}{\partial Z}\right) - \frac{\bar{T}}{\bar{P}}\frac{\partial}{\partial Z}\left(\frac{\bar{P}}{\bar{T}}y_i\bar{v}\right) - \psi\frac{\bar{T}}{\bar{P}}\frac{\partial x_i}{\partial \tau} - \frac{y_i}{\bar{P}}\frac{\partial \bar{P}}{\partial \tau} + \frac{y_i}{\bar{T}}\frac{\partial \bar{T}}{\partial \tau} \tag{S1}$$

Total mass balance:

$$\frac{\partial \bar{P}}{\partial \tau} = \bar{T}\frac{\partial}{\partial Z}\left(\frac{\bar{P}}{\bar{T}}\bar{v}\right) - \psi\bar{T}\sum_{i=1}^{N}\frac{\partial x_i}{\partial \tau} + \frac{\bar{P}}{\bar{T}}\frac{\partial \bar{T}}{\partial \tau} \tag{S2}$$

Mass transfer rate equation:

$$\frac{\partial x_i}{\partial \tau} = \alpha_i({x_i}^* - x_i) \tag{S3}$$

Energy balance:

$$\frac{\partial \bar{T}}{\partial \tau} = \Omega_1\frac{\partial^2 \bar{T}}{\partial Z^2} - \Omega_2\left(\frac{\partial}{\partial Z}(\bar{v}\bar{P}) + \frac{\partial \bar{P}}{\partial \tau}\right) - \Omega_4\bar{T}\sum_{i=1}^{N}\frac{\partial x_i}{\partial \tau} - \sum_{i=1}^{N}\Omega_{3i}\frac{\partial x_i}{\partial \tau} - \Omega_5(\bar{T} - \bar{T}_W) \tag{S4}$$

Wall temperature balance:

$$\frac{\partial \bar{T}_W}{\partial \tau} = \prod_1\frac{\partial^2 \bar{T}_W}{\partial Z^2} + \prod_2(\bar{T} - \bar{T}_W) - \prod_3(\bar{T}_W - \bar{T}_a) \tag{S5}$$

Frictional pressure drop

$$\frac{-d\bar{P}}{dz} = 180\frac{\mu L v_0}{P_0 d_p^2}\left(\frac{1-\varepsilon_b}{\varepsilon_b}\right)^2 \qquad \text{(S6)}$$

The dimensionless groups in Equations (S1) -(S6) are given in Table S1. Each of the equations requires appropriate initial and boundary conditions, which are given in Table S2. The initial conditions of each step are the final conditions of the previous step

Table S1: Dimensionless groups in the model equations.

$$Pe = \frac{v_o L}{D_L} \quad Pe_H = \frac{\varepsilon_b u_o L \rho_g C_{pg}}{K_z} \quad \psi = \frac{1-\varepsilon_b}{\varepsilon_b}\frac{R T_o q_s}{P_0} \quad \alpha_i = \frac{k_i L}{v_0} \quad \Pi_1 = \frac{K_w}{\rho_w C_{pw} v_0 L} \quad \Pi_2 = \frac{2 r_i h_i L}{\rho_w C_{pw} v_o \left(r_o^2 - r_i^2\right)}$$

$$\Pi_3 = \frac{2 r_o h_o L}{\rho_w C_{pw} v_o \left(r_o^2 - r_i^2\right)} \quad \Omega_1 = \frac{K_z}{\left(\frac{1-\varepsilon_b}{\varepsilon_b}\left(\rho_s c_{ps} + c_{pa} q_s \sum_{i=1}^{n} x_i\right)\right)\varepsilon_b v_o L} \quad \Omega_2 =$$

$$\frac{C_{pg}}{\left(\frac{1-\varepsilon_b}{\varepsilon_b}\left(\rho_s c_{ps} + c_{pa} q_s \sum_{i=1}^{n} x_i\right)\right)}\frac{P_0}{R T_0} \quad \Omega_{3i} = \frac{\frac{1-\varepsilon_b}{\varepsilon_b}\left(-\Delta H_i\right) q_s}{T_0\left(\frac{1-\varepsilon_b}{\varepsilon_b}\left(\rho_s c_{ps} + c_{pa} q_s \sum_{i=1}^{n} x_i\right)\right)}$$

$$\Omega_4 = \frac{\frac{1-\varepsilon_b}{\varepsilon_b} C_{pa} q_s}{\left(\frac{1-\varepsilon_b}{\varepsilon_b}\left(\rho_s c_{ps} + c_{pa} q_s \sum_{i=1}^{n} x_i\right)\right)} \quad \Omega_5 = \frac{2 h_i L}{\varepsilon_b r_i v_o \left(\frac{1-\varepsilon_b}{\varepsilon_b}\left(\rho_s c_{ps} + c_{pa} q_s \sum_{i=1}^{n} x_i\right)\right)}$$

Table S2: Boundary conditions for a 6-step VSA process.

**Step**	Inlet BCs	Outlet BCs
**Adsorption**	$\frac{1}{Pe}\frac{\partial y_i}{\partial Z}\Big\vert_{Z=0} = -\bar{v}\vert_{Z=0}(y_{feed} - y_i\vert_{Z=0})$<br>$\bar{v}\vert_{Z=0} = 1$<br>$\frac{1}{Pe_h}\frac{\partial \bar{T}}{\partial Z}\Big\vert_{Z=0} = -\bar{v}\vert_{Z=0}(\bar{T}_{feed} - \bar{T}\vert_{Z=0})$<br>$\bar{T}_W\vert_{Z=0} = \bar{T}_a$	$\frac{\partial y_i}{\partial Z}\Big\vert_{Z=1} = 0$<br>$\bar{P}\vert_{Z=1} = 1$<br>$\frac{\partial \bar{T}}{\partial Z}\Big\vert_{Z=1} = 0$<br>$\bar{T}_W\vert_{Z=1} = \bar{T}_a$
**Co-current evacuation**	$\frac{\partial y_i}{\partial Z}\Big\vert_{Z=0} = 0$<br>$\bar{v}\vert_{Z=0} = 0$<br>$\frac{\partial \bar{T}}{\partial Z}\Big\vert_{Z=0} = 0$<br>$\bar{T}_W\vert_{Z=0} = \bar{T}_a$	$\frac{\partial y_i}{\partial Z}\Big\vert_{Z=1} = 0$<br>$\frac{\partial \bar{T}}{\partial Z}\Big\vert_{Z=1} = 0$<br>$\bar{v}\vert_{Z=1} = \frac{F_{pump}}{v_0 A\varepsilon}$<br>$\bar{T}_W\vert_{Z=1} = \bar{T}_a$
**Counter current-Evacuation**	$\frac{\partial y_i}{\partial Z}\Big\vert_{Z=0} = 0$<br>$\bar{v}\vert_{Z=1} = \frac{F_{pump}}{v_0 A\varepsilon}$<br>$\frac{\partial \bar{T}}{\partial Z}\Big\vert_{Z=0} = 0$<br>$\bar{T}_W\vert_{Z=0} = \bar{T}_a$	$\frac{\partial y_i}{\partial Z}\Big\vert_{Z=1} = 0$<br>$\bar{v}\vert_{Z=1} = 0$<br>$\frac{\partial \bar{T}}{\partial Z}\Big\vert_{Z=1} = 0$<br>$\bar{T}_W\vert_{Z=1} = \bar{T}_a$
**Light product pressurization**	$\frac{\partial y_i}{\partial Z}\Big\vert_{Z=0} = 0$<br>$\bar{v}\vert_{Z=0} = 0$<br>$\frac{\partial \bar{T}}{\partial Z}\Big\vert_{Z=0} = 0$<br>$T_W\vert_{Z=0} = \bar{T}_a$	$\frac{1}{Pe}\frac{\partial y_i}{\partial Z}\Big\vert_{Z=1} = -\bar{v}\vert_{Z=1}(y_{exit,adsorption} - y_i\vert_{Z=1})$<br>$\bar{v}\vert_{Z=1} = \frac{\bar{v}_{exit,adsorption}\bar{P}_{exit,adsorption}}{\bar{P}_{Z=1}}\frac{\bar{T}_{exit,adsor}}{\bar{T}_{Z=1}}$ [illegible]<br>$\frac{1}{Pe_h}\frac{\partial \bar{T}}{\partial Z}\Big\vert_{Z=1} = -\bar{v}\vert_{Z=1}(\bar{T}_{exit,adsorption} - \bar{T}\vert_{Z=1})$<br>$\bar{T}_W\vert_{Z=1} = \bar{T}_a$

**Heavy reflux**	$\frac{1}{Pe}\frac{\partial y_i}{\partial Z}\Big\vert_{Z=0} = -\bar{v}\big\vert_{Z=0}(y_{exit,Lightreflux} - y_i\vert_{Z=0})$<br>$\bar{v}\vert_{Z=0} = \frac{\bar{v}_{exit,lightreflux}\bar{P}_{exit,lightreflux}}{\bar{P}_{Z=0}}\frac{\bar{T}_{exit,lightreflux}}{\bar{T}_{Z=0}}$<br>$\frac{1}{Pe_h}\frac{\partial \bar{T}}{\partial Z}\Big\vert_{Z=0} = -\bar{v}\big\vert_{Z=0}(\bar{T}_{lightreflux} - \bar{T}\vert_{Z=0})$<br>$\bar{T}_W\big\vert_{Z=0} = \bar{T}_a$	$\frac{\partial y_i}{\partial Z}\Big\vert_{Z=1} = 0$<br>$\bar{P}\big\vert_{Z=1} = 1$<br>$\frac{\partial \bar{T}}{\partial Z}\Big\vert_{Z=1} = 0$<br>$\bar{T}_W\big\vert_{Z=1} = \bar{T}_a$
**Light Reflux/purge**	$\frac{\partial y_i}{\partial Z}\Big\vert_{Z=0} = 0$<br>$\bar{P}\big\vert_{Z=0} = \frac{P_L}{P_0}$<br>$\frac{\partial \bar{T}}{\partial Z}\Big\vert_{Z=0} = 0$<br>$\bar{T}_W\big\vert_{Z=0} = \bar{T}_a$	$\frac{1}{Pe}\frac{\partial y_i}{\partial Z}\Big\vert_{Z=1} = -\bar{v}\big\vert_{Z=1}(y_{exit,adsorption} - y_i\vert_{Z=1})$<br>$\bar{v}\vert_{Z=1} = \frac{\bar{v}_{exit,adsorption}\bar{P}_{exit,adsorption}}{\bar{P}_{Z=1}}\frac{\bar{T}_{exit,adsor}}{\bar{T}_{Z=1}}$<br>$\frac{1}{Pe_h}\frac{\partial \bar{T}}{\partial Z}\Big\vert_{Z=1} = -\bar{v}\big\vert_{Z=1}(\bar{T}_{exit,adsorption} - \bar{T}\vert_{Z=1})$<br>$\bar{T}_W\big\vert_{Z=1} = \bar{T}_a$

Correlations used to calculate parameters

Heat of adsorption

$$\Delta H_i = \frac{q_{s1,i}b_{1,i}\left(\Delta U_{1,i} - RT_0\right) + q_{s2,i}b_{2,i}\left(\Delta U_{2,i} - RT_0\right)}{q_{s1,i}b_{1,i} + q_{s2,i}b_{2,i}} \quad \text{(S7)}$$

Specific heat capacity of gas mixtures

$$C_{p,gas,mix} = \sum_{i=1}^{n} y_{i,feed} C_P^i \quad \text{(S8)}$$

For packed bed the axial dispersion was calculated according to the equation below

$$D_L = \frac{20 D_{mol}}{\varepsilon} + 0.5 v_0 d_P \quad \text{(S9)}$$

D_{mol} is the molecular diffusivity, d_P is the pellet diameter and v_0 is the interstitial velocity

Molecular diffusion

$$D_{mol} = \frac{1.86x10^{-7} T^{1.5} \left(\frac{1}{M_1} + \frac{1}{M_2} \right)^{0.5}}{P \sigma_{1,2}^2 \Omega} \quad \text{(S10)}$$

Kundsen diffusion corrected by Derjaguin correction factor

$$D_K = 0.672 r_{pore} \left(\frac{T}{M} \right)^{0.5} \quad \text{(S11)}$$

r_{pore} is the macropore radii and T and M are temperature in K and molecular weight of the gas.

The macropore diffusivity is given by

$$D_{macro} = \frac{D_{Mol} D_K}{D_{mol} D_K} \quad \text{(S12)}$$

Axial thermal conductivity

$$k_z = k_g \left(5 + 0.1 \mathrm{Re} \, \mathrm{Pr} \right) \quad \text{(S13)}$$

k_g is the thermal conductivity of the gas mixture Pr number taken 0.7 for gases.

The LDF coefficient for the packed bed was calculated as

$$k_{LDF} = \frac{15 \varepsilon_P \frac{D_{macro}}{\tau}}{0.25 d_P^2 \frac{dq}{dc}} \quad \text{(S14)}$$

The thermal conductivity and viscosity of the gas mixtures are provided as following

$$k_{g,gas,mix} = \sum_{i=1}^{n} y_{i,feed} k_g^i \quad \text{(S15)}$$

$$\mu_{gas,mix} = \sum_{i=1}^{n} y_{i,feed} \mu_i \quad \text{(S16)}$$

The energy consumption by the vacuum pumps, blowers and compressors are provided below

$$Energy_{vacuum} = \varepsilon \pi r_i^2 \frac{\gamma}{\gamma - 1} \int_{t=0}^{t=t_{vacuum}} vP \left[\frac{1}{\eta\left(P(t)_{vacuum}\right)} \left(\frac{P_{atm}}{P(t)_{vacuum}} \right)^{\frac{\gamma}{\gamma-1}} - 1 \right] dt \qquad \text{(S17)}$$

$$Energy_{compress} = \frac{1}{\eta} \varepsilon \pi r_i^2 \frac{\gamma}{\gamma - 1} \int_{t=0}^{t=t_{vacuum}} vP \left[\left(\frac{\overline{P}(t)_{in}}{P_{atm}} \right)^{\frac{\gamma}{\gamma-1}} - 1 \right] dt \qquad \text{(S18)}$$

In equation S16, the efficiency of the vacuum pump η is a function of pressure (Subraveti et al., 2021)

$$\eta = \frac{15.84P}{1+19.8P} \qquad \text{(S19)}$$

Table S3: CO_2 and N_2 isotherms measured on the pellets by Gravimetry

**CO_2**		**N_2**	
**293 K**			
P(bar)	q(mol/kg)	P(bar)	q(mol/kg)
0.021027	0.1711927	0.08468	0.006415
0.082011	0.6995299	0.2086	0.018966
0.1268	1.2285697	0.305869	0.026148
0.143915	1.3930609	0.407539	0.03439
0.211855	1.8932366	0.528371	0.04416
0.305825	2.3047707	0.642204	0.052745
0.432038	2.6404051	0.751399	0.061517
0.5845	2.8974978	0.853377	0.068117
0.762074	3.0834512	0.927049	0.072667
0.98226	3.2454219	0.99954	0.07823
**303 K**			
P(bar)	q(mol/kg)	P(bar)	q(mol/kg)
0.045238	0.2598693	0.092205	0.006488
0.122716	0.6867689	0.203412	0.014826
0.176	1.1000276	0.311775	0.022286
0.231385	1.5126264	0.41677	0.029482
0.298626	1.8800569	0.522159	0.036089
0.392771	2.1922618	0.621948	0.042573
0.505204	2.4440547	0.734513	0.049532
0.64826	2.6573124	0.83614	0.05599
0.80431	2.8263136	0.926437	0.062146
0.987747	2.9801045	1.010215	0.067905
**313 K**			

P(bar)	q(mol/kg)	P(bar)	q(mol/kg)
0.033408	0.1293688	0.086605	0.005379
0.118935	0.4201573	0.214524	0.012816
0.199168	0.6757865	0.323631	0.019307
0.271308	0.9455053	0.42552	0.025004
0.336361	1.2352883	0.530865	0.031468
0.402683	1.5396211	0.643385	0.038484
0.500241	1.8387543	0.744312	0.045022
0.622605	2.0768696	0.831765	0.050316
		0.932824	0.056777
		1.011921	0.061774

Table S4: CO_2 and N_2 isotherms generated by molecular simulations

**CO_2**		**N_2**	
298K		298K	
P(bar)	q(mol/kg)	P(bar)	q(mol/kg)
0.000	0.000	0.000	0.000
0.000	0.000	0.001	0.000
0.000	0.002	0.005	0.001
0.001	0.020	0.010	0.003
0.005	0.099	0.020	0.006
0.010	0.196	0.030	0.009
0.020	0.394	0.040	0.012
0.030	0.579	0.050	0.015
0.040	0.741	0.060	0.018
0.050	0.899	0.070	0.022
0.060	1.026	0.080	0.025
0.070	1.158	0.090	0.028
0.080	1.276	0.100	0.031
0.090	1.363	0.110	0.034
0.100	1.489	0.120	0.037
0.110	1.570	0.130	0.040
0.120	1.672	0.140	0.043
0.130	1.730	0.150	0.046
0.140	1.765	0.160	0.049
0.150	1.841	0.170	0.052
0.160	1.892	0.180	0.055
0.170	1.939	0.190	0.058
0.180	1.974	0.200	0.061
0.190	2.030	0.250	0.076
0.200	2.062	0.300	0.090
0.250	2.229	0.350	0.105
0.300	2.360	0.400	0.118

0.350	2.452	0.450	0.133
0.400	2.517	0.500	0.146
0.450	2.586	0.550	0.160
0.500	2.625	0.600	0.173
0.550	2.680	0.650	0.188
0.600	2.720	0.700	0.200
0.650	2.769	0.750	0.213
0.700	2.782	0.800	0.226
0.750	2.811	0.850	0.239
0.800	2.831	0.900	0.251
0.850	2.856	0.950	0.262
0.900	2.880	1.000	0.277
0.950	2.905		
1.000	2.934		
308K		308K	
P(bar)	q(mol/kg)	P(bar)	q(mol/kg)
0.000	0.000	0.000	0.000
0.000	0.000	0.001	0.000
0.000	0.001	0.005	0.001
0.001	0.012	0.010	0.002
0.005	0.064	0.020	0.005
0.010	0.128	0.030	0.007
0.020	0.250	0.040	0.009
0.030	0.371	0.050	0.012
0.040	0.477	0.060	0.014
0.050	0.597	0.070	0.017
0.060	0.699	0.080	0.019
0.070	0.802	0.090	0.021
0.080	0.885	0.100	0.024
0.090	0.982	0.110	0.026
0.100	1.059	0.120	0.028
0.110	1.152	0.130	0.031
0.120	1.213	0.140	0.033
0.130	1.264	0.150	0.036
0.140	1.342	0.160	0.038
0.150	1.382	0.170	0.040
0.160	1.459	0.180	0.042
0.170	1.495	0.190	0.045
0.180	1.573	0.200	0.047
0.190	1.609	0.250	0.058
0.200	1.630	0.300	0.070
0.250	1.846	0.350	0.081
0.300	1.978	0.400	0.092
0.350	2.105	0.450	0.103

0.400	2.186	0.500	0.114
0.450	2.303	0.550	0.125
0.500	2.359	0.600	0.136
0.550	2.440	0.650	0.146
0.600	2.454	0.700	0.157
0.650	2.515	0.750	0.168
0.700	2.545	0.800	0.177
0.750	2.595	0.850	0.188
0.800	2.619	0.900	0.197
0.850	2.631	0.950	0.208
0.900	2.686	1.000	0.217
0.950	2.710		
1.000	2.741		
318K		318K	
P(bar)	q(mol/kg)	P(bar)	q(mol/kg)
0.000	0.000	0.000	0.000
0.000	0.000	0.001	0.000
0.000	0.001	0.005	0.001
0.001	0.008	0.010	0.002
0.005	0.041	0.020	0.004
0.010	0.082	0.030	0.006
0.020	0.162	0.040	0.007
0.030	0.242	0.050	0.009
0.040	0.320	0.060	0.011
0.050	0.397	0.070	0.013
0.060	0.467	0.080	0.015
0.070	0.538	0.090	0.017
0.080	0.606	0.100	0.019
0.090	0.673	0.110	0.020
0.100	0.734	0.120	0.022
0.110	0.811	0.130	0.024
0.120	0.864	0.140	0.026
0.130	0.905	0.150	0.028
0.140	0.977	0.160	0.030
0.150	1.028	0.170	0.032
0.160	1.080	0.180	0.033
0.170	1.123	0.190	0.035
0.180	1.164	0.200	0.037
0.190	1.185	0.250	0.046
0.200	1.271	0.300	0.055
0.250	1.440	0.350	0.064
0.300	1.614	0.400	0.073
0.350	1.740	0.450	0.082
0.400	1.837	0.500	0.091

0.450	1.933	0.550	0.100
0.500	2.014	0.600	0.108
0.550	2.071	0.650	0.117
0.600	2.141	0.700	0.125
0.650	2.203	0.750	0.134
0.700	2.230	0.800	0.142
0.750	2.293	0.850	0.150
0.800	2.345	0.900	0.159
0.850	2.389	0.950	0.167
0.900	2.417	1.000	0.174
0.950	2.474		
1.000	2.455		

Table S5: Input parameters to the simulation

Input parameters	Value
Bed length (m)	1
Internal diameter (m)	0.289
External diameter(m)	0.324
Isentropic efficiency of the blower (%)	72
Sorbent density (kg/m^3)	1075.4
Sorbent specific heat (J/kg K)	1000
Gas viscosity (Pa s)	1.7×10^{-5}
Knudsen diffusivity at 298 K (m^2/s)	2.8×10^{-5}
Molecular diffusivity at 298 K (m^2/s)	1.65×10^{-5}
Internal and external heat transfer coefficient (W/m^2 K)	0
Pellet porosity	0.378
Tortuosity	2.5
Wall density (kg/m^3)	7800
Wall specific heat (J/kg K)	502

Table S6 Operating conditions corresponding to the minimum energy and maximum productivity values in the pareto

Isotherms/conditions		Adsorption time (s)	reflux time (s)	Co evac pressure (bar)	Cn-evac pressure (bar)	V_0 (m/s)	Co evac pump (m^3/hr)	cn-evac pump (m^3/hr)
Min Energy	Experiment	48.74	13.95	0.43	0.19	1.1	56.9	115.3
	Simulation	22.01	9.77	0.22	0.11	1.42	76.2	408.3
Max Prod	Experiment	48.3	14.8	0.45	0.16	1.37	57.3	117.6
	Simulation	23.2	10.8	0.22	0.13	1.43	86.7	367.4

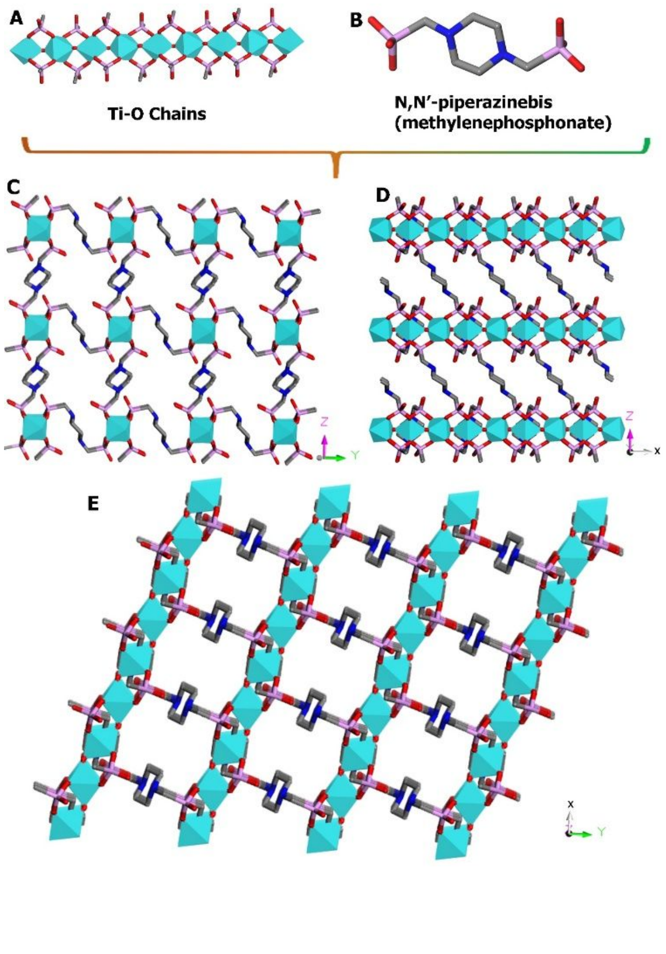

Figure S1: Crystal structure of MIL-91(Ti). (A) and (B) represent the Ti-O chains and the linker, respectively. (C-E) view of the MOF from different axis.

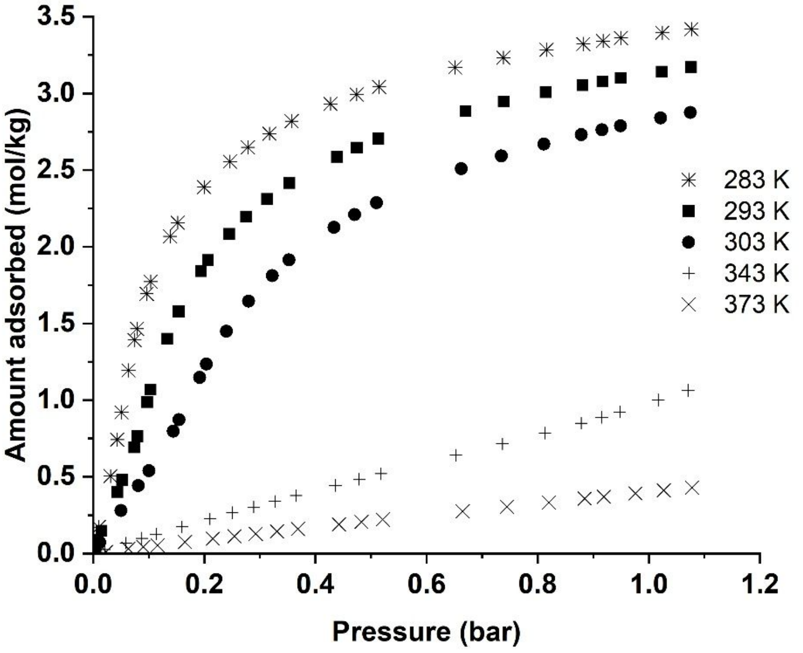

Figure S2: CO_2 isotherms measured by the BELSORP MAX G volumetric apparatus

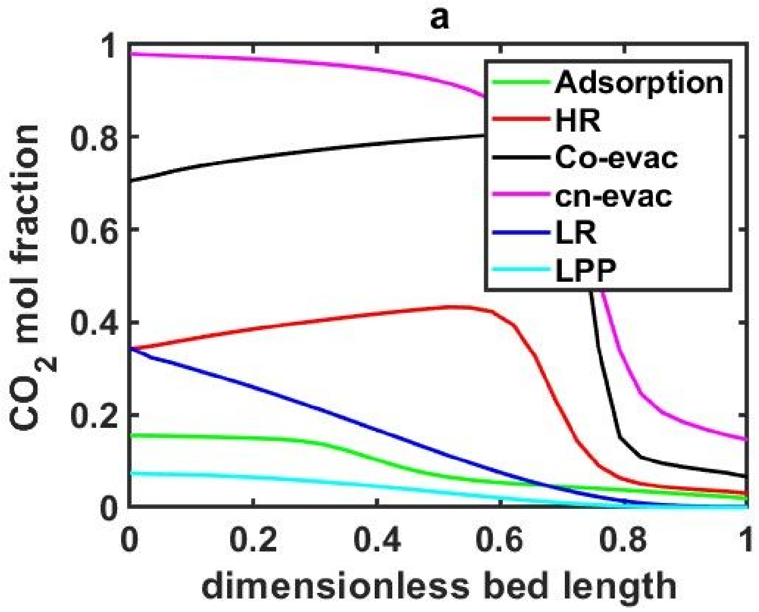

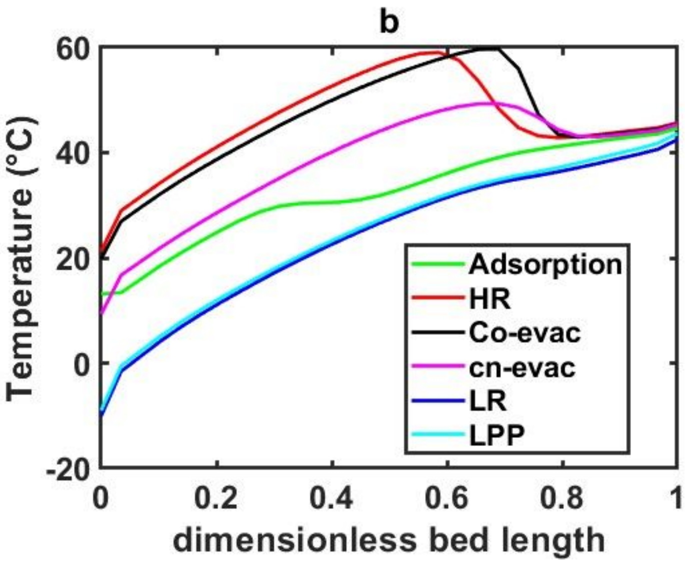

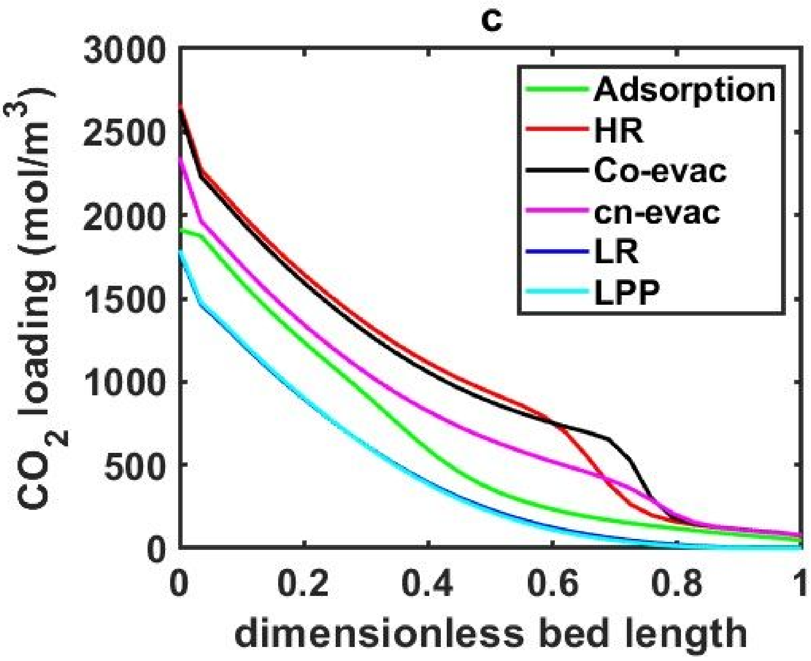

Figure S3: Cyclic steady state profile for (a) gas phase CO_2 (b) temperature and (c) solid phase CO_2 for the minimum energy conditions with the experimental isotherms.

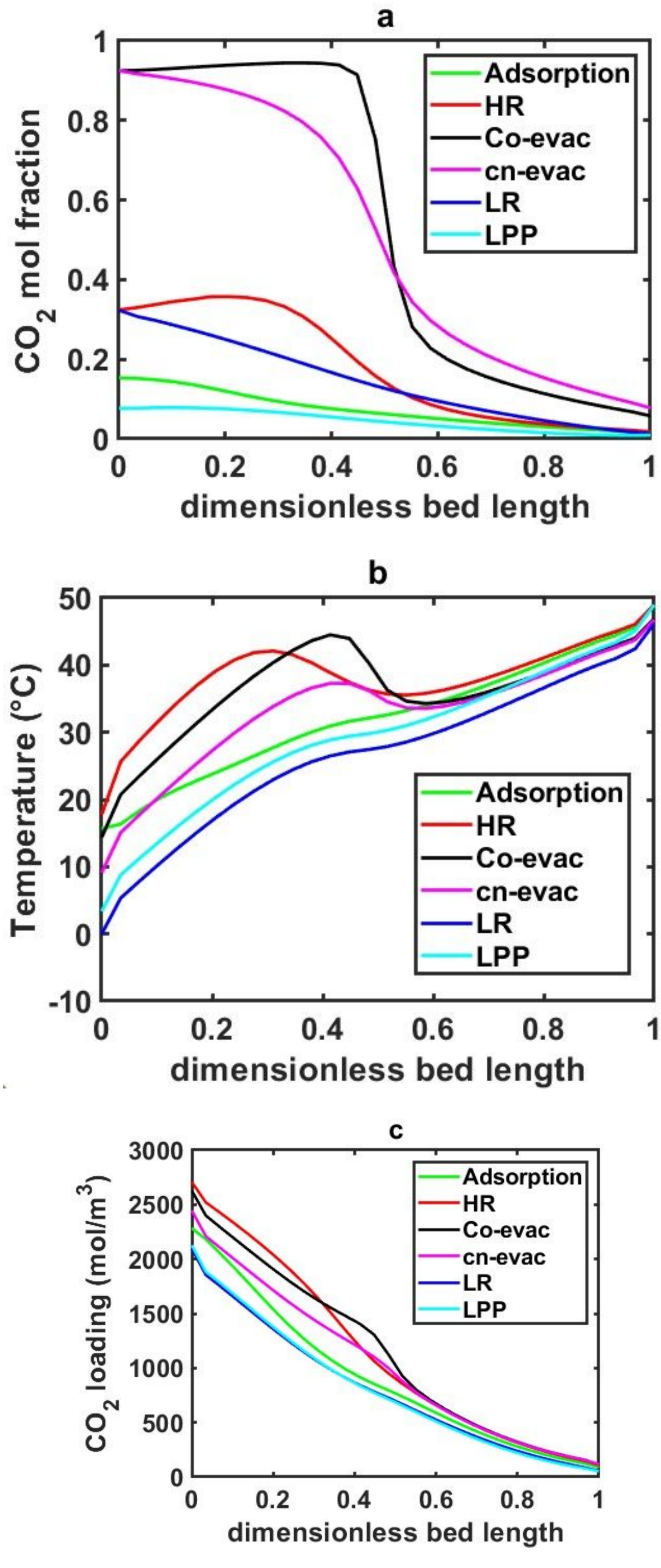

Figure S4: Cyclic steady state profile for (a) gas phase CO_2 (b) temperature and (c) solid phase CO_2 for the minimum energy conditions with the simulated isotherms.

Notations

b_0: Affinity coefficient of the dual-site Langmuir isotherm for sites 1 and 2

$C_{T:}$ Total gas phase concentration (mol/m^3)

$c_{i:}$ Gas phase concentration of component i (mol/m^3)

$C_{pg:}$ Specific heat capacity of the gas mixture (J/kg K)

$C_{pa:}$ Specific heat capacity of the adsorbed phase (J/kg K)

$C_{ps:}$ Specific heat capacity of the adsorbent (J/kg K)

$C_{pw:}$ Specific heat capacity of the column wall (J/kg K)

$D_{L:}$ axial dispersion coefficient (m^2/s)

ΔH: Heat of adsorption (J/mol)

h_i: internal heat transfer coefficient (W/m^2 K)

h_0: internal heat transfer coefficient (W/m^2 K)

k_z: Axial thermal conductivity of the gas (W/m K)

k_w: Thermal conductivity of the wall (W/m K)

$k_{LDF\,i}$: Linear driving force coefficient (s^{-1})

P: Total pressure in the system (Pa)

P_H: High pressure in the adsorption step (Pa)

P_L: Vacuum pressure in the counter-current evacuation step (Pa)

P_{INT}: Vacuum pressure in the co-current evacuation step (Pa)

P_{ATM}: Ambient pressure (Pa)

q_i: Solid phase concentration (mol/m^3)

q_s: Solid phase concentration at saturation (mol/m^3)

q_i^*: Equilibrium solid phase concentration at saturation (mol/m^3)

R: Gas constant (J/mol/K)

r_i: Column internal radius (m)

r_0: Column external radius (m)

r_p: Pellet radius (m)

T: Temperature inside the column (K)

T_W: wall temperature (K)

T_a: Ambient temperature (K)

t: time (s)

ΔU: Internal energy of adsorption (J/mol)

v: interstitial velocity (m/s)

y_i: mol fraction of component i

z: Axial dimension (m)

Greek symbols

ε: bed void fraction

ε_P: bed void fraction

ρ_s: density of the adsorbent (kg/m^3)

ρ_g: density of the gas mixture (kg/m^3)

ρ_w: density of the wall (kg/m^3)

η: vacuum pump efficiency

Υ: ratio of the specific heats

μ: viscosity of gas mixture (Pa s)

References:

Subraveti, S.G., Roussanaly, S., Anantharaman, R., Riboldi, L., 2021. Techno-economic assessment of optimised vacuum swing adsorption for post-combustion CO_2 capture from stream-methane reformer flue gas. Sep. Purif. Technol. 256, 117832. https://doi.org/10.1016/j.seppur.2020.117832.
